# Cytotoxicity, early safety screening, and antimicrobial potential of minor oxime constituents of essential oils and aromatic extracts

**DOI:** 10.1038/s41598-022-09210-z

**Published:** 2022-03-29

**Authors:** Alicja Karolina Surowiak, Marta Sowała, Michał Talma, Katarzyna Groborz, Lucyna Balcerzak, Stanisław Lochyński, Daniel Jan Strub

**Affiliations:** 1 Department of Chemical Biology and Bioimaging, Faculty of Chemistry, Wrocław University of Science and Technology, Wyb. Wyspiańskiego 27, 50-370 Wrocław, Poland; 2 Department of Bioorganic Chemistry, Faculty of Chemistry, Wrocław University of Science and Technology, Wyb. Wyspiańskiego 27, 50-370 Wrocław, Poland; 3 Institute of Cosmetology, Wroclaw College of Physiotherapy, T. Kościuszki 4, 50-038 Wrocław, Poland; 4 Liquid Technologies SP. Z O.O., Gdańska 13, 50-344 Wrocław, Poland

**Keywords:** Antimicrobials, Natural products

## Abstract

Due to market and legislative expectations, there is a constant need to explore new potential antimicrobial agents for functional perfumery. In this study, we evaluated the antimicrobial activity of 53 low molecular oximes and the corresponding carbonyl compounds against *Escherichia coli, Enterococcus hirae, Pseudomonas aeruginosa, Bacillus cereus, Staphylococcus aureus, Aspergillus brasiliensis, Legionella pneumophila* and *Candida albicans*. The most potent compound was α-isomethylionone oxime, which exhibited a minimum inhibitory concentration (MIC) of 18.75 µg/mL against *E. hirae*. The evaluation of the MICs for bacterial and fungal strains was performed for selected compounds, for example, the MIC of 2-phenylpropionaldehyde, *cis*-jasmone oxime, and *trans*-cinnamaldehyde measured against *A. brasiliensis* was 37.50 µg/mL. ADME-Tox (Absorption, Distribution, Metabolism, Excretion, and Toxicity) and 3-(4,5-dimethylthiazol-2-yl)-5-(3-carboxymethoxyphenyl)-2-(4-sulfophenyl)-2*H*-tetrazolium (MTS) cell viability assays were performed to assess the cytotoxicity of tested compounds. ADME-Tox indicated the safety and promising properties of selected compounds, which enables their usage as nontoxic supporting antibacterial agents. The results of the in vitro MTS assay were consistent with the ADME-Tox results. None of the compounds tested was toxic to Human Embryonic Kidney 293T (HEK293T) cells, with all cell viabilities exceeding 85%.

## Introduction

Despite the number of currently known volatile organic compounds, many of them cannot be used in various areas of human activity due to their toxic effects. Chemical regulations, such as REACH (Registration, Evaluation, Authorization and Restriction of Chemicals), CPSIA (Consumer Product Safety Improvement Act) and RoHS (Restriction of Hazardous Substances Directive), were created to protect the environment and the welfare of humans^1,2^. New chemical ingredients for cosmetics and household chemicals must be proven safe before being put on the EU market. New product development in the flavor and fragrance (F&F) industry is an expensive process. Therefore, it is necessary to perform preliminary cost-effective evaluations that would allow minimizing risks. One of such tools is an in-silico ADME-Tox which is a simple and inexpensive method for optimization in pharmaceutical lead discovery. Moreover, this approach predicts how chemical compounds act and preliminarily assess whether they are safe and effective^3^. ADME-Tox improves the selection of molecules of interest by high-throughput subset selection^4^ and eliminates compounds with toxicity^5^. ADME-Tox predicts particular druglike properties on the basis of chemical structure. In silico toxicity predictions are validated in the next step by the in vitro cell viability assay, such as MTS assay. In vitro cytotoxicity assay is a valuable method in human health risk assessment, wherein its accuracy, sensitivity, and simplicity can provide a rapid indication of the toxicity of tested compound^6^.

Volatile organic compounds are used in flavor and fragrance, food, perfumery, cosmetics and toiletries, fine chemicals, and pharmaceutical industries^7^. Carbonyl compounds derived from essential oils are known to possess antimicrobial effects^8^. Oximes and their derivatives are one of the interesting groups of minor secondary metabolites of plants, yet they have not been extensively studied in this matter^9–11^. The primary concern in applying low-molecular weight carbonyl compounds in functional product compositions is their instability and propensity to oxidation^12^. A solution to that inconvenience could be the application of the corresponding oximes, which are characterized by high hydrolytic stability^13^. The research carried out so far focused mainly on synthetic aspects of oximes^14,15^, and there are only few references related to the antimicrobial activity of this class of compounds. Some higher molecular weight oximes were tested on some microorganisms and were found to be effective, yet the mechanism of the observed antimicrobial activity is still not entirely clear^16^.

This study is a continuation of our search for new ingredients for functional perfumery. We have recently reported a thorough study on sensory properties of low molecular oximes, which are minor constituents of essential oils and aromatic extracts^10^. We have identified several of them that could be an interesting addition to fragrance compositions. In this work, our aim was to explore the underdeveloped niche and assess the potential usefulness of volatile oximes as antimicrobials in relevant strains that reside in households, industrial and hospital air conditioning systems, such as *E. coli, E. hirae, P. aeruginosa, B. cereus, S. aureus, A. brasiliensis, L. pneumophila,* and *C. albicans*. The microorganisms were selected based on the standard for chemical disinfectants and antiseptics (EN 13697:2019). These products might be the target application for test compounds. Cytotoxicity and early safety screenings were performed using ADME-Tox (estimation of caucasian colon adenocarcinoma (caco-2) cell permeability, Madin-Darby Canine Kidney (MDCK) cell permeability, brain/blood coefficient, binding to human albumin, skin permeability, oral adsorption, and other parameters) and MTS assay (with HEK293T cell lines).

## Results

### ADME-Tox

All compounds fulfilled Lipinski’s rule of five^17^ (Molecular Weight < 500, predicted octanol/water partition coefficient (QPlogPo/w) < 5, estimated number of donated hydrogen bonds (donorHB) ≤ 5, estimated number of accepted hydrogen bonds (accptHB) (≤ 10), but two of them were negative for Jorgensen’s rule of three^18^ predicted aqueous solubility (QPlogS) > − 5.7, predicted apparent Caco-2 cell permeability for nonactive transport (QPPCaco) > 22 nm/s, Primary Metabolites < 7): the geranyl acetone and its oxime. Despite this, they were included for further evaluation. Most of the predictions were in the recommended range (according to the QikPro 3.5 user manual), although the reverse phenomenon was seen for the partition coefficient for octanol and water. Great Caco-2 cell permeability was demonstrated for all compounds. None of the compounds exhibited poor MDCK cell permeability; however, not all (n = 73) exhibited high scores (< 500). Only two oximes and one aldehyde were considered out of the recommended range as blockers for HERG K^+^ channels. All compounds were in the proper range for the brain-blood partition coefficient. Table 1 shows the results for the seven most potent compounds. For the remaining data, see the Tables S4 and S5 .

**Table 1:** Properties of ADME-Tox for the selected compounds with the lowest MIC.

Compound	MW	SASA	donorHB	accptHB	QPlogPo/w	QPlogHERG	QPPCaco	QPlogBB	QPPMDCK
α-Isomethylionone oxime	221	488	1.00	2.70	2.98	− 3.34	2040	− 0.35	1070
Pseudoionone oxime	207	540	1.00	2.70	3.25	− 4.50	1840	− 0.66	958
β-Ionone oxime	207	501	1.00	2.70	2.82	− 3.86	2000	− 0.40	1040
*trans*-Cinnamaldehyde oxime	147	378	1.00	3.20	1.29	− 4.43	1320	− 0.51	668
2-Phenylpropionaldehyde	134	356	0.00	2.00	1.87	− 3.68	2570	− 0.04	1370
α-Hexylcinnamaldehyde oxime	231	565	1.00	3.20	3.52	− 5.44	1830	− 0.81	952
*cis*-Jasmone oxime	179	446	1.00	2.70	2.34	− 3.56	2460	− 0.28	1310

Compound	QPpolrz	QPlogPC16	QPlogPoct	QPlogPw	QPlogS	CIQPlogS	QPlogKp	QPlogKhsa	% Oral Abs
α-Isomethylionone oxime	24.8	7.04	10.2	4.45	− 3.38	− 2.72	− 2.35	0.21	100
Pseudoionone oxime	24.9	7.46	10.1	4.21	− 3.84	− 2.34	− 2.01	0.18	100
β-Ionone oxime	24.4	6.78	10.0	4.51	− 3.53	− 2.44	− 2.36	0.17	100
*trans*-Cinnamaldehyde oxime	16.5	6.16	8.07	6.43	− 1.37	− 1.40	− 1.88	− 0.56	90.4
2-Phenylpropionaldehyde	16.6	5.24	6.17	3.77	− 1.60	− 1.35	− 1.82	− 0.38	100
α-Hexylcinnamaldehyde oxime	27.0	9.07	11.2	5.44	− 3.79	− 2.99	− 1.24	0.12	100
*cis*-Jasmone oxime	20.4	5.94	8.67	4.45	− 2.65	− 1.96	− 2.11	− 0.10	100

*MW* molecular weight, *SASA* total solvent-accessible surface area, *donorHB* estimated number of donated hydrogen bonds, *accptHB* estimated number of accepted hydrogen bonds, *QPlogPo/w* predicted octanol/water partition coefficient, *QPpolrz* predicted polarizability in cubic angstroms, *QPlogPC16* predicted hexadecane/gas partition coefficient, *QPlogPoct* predicted octanol/gas partition coefficient, *QPlogPw* predicted water/gas partition coefficient, *QPlogS* predicted aqueous solubility, *CIQPlogS* conformation-independent predicted aqueous solubility, *QPlogHERG* predicted IC_50_ value for blockage of HERG K+ channels, *QPPCaco* predicted apparent Caco-2 cell permeability for non-active transport, *QPlogBB* predicted brain/blood partition coefficient, QPPMDCK− predicted apparent MDCK cell permeability for non-active transport, *QPlogKp* predicted skin permeability, *QPlogKhsa* prediction of binding to human serum albumin, *Percent Human Oral Absorption* predicted human oral absorption on 0–100% scale.

### MTS assay

**Figure 1:**
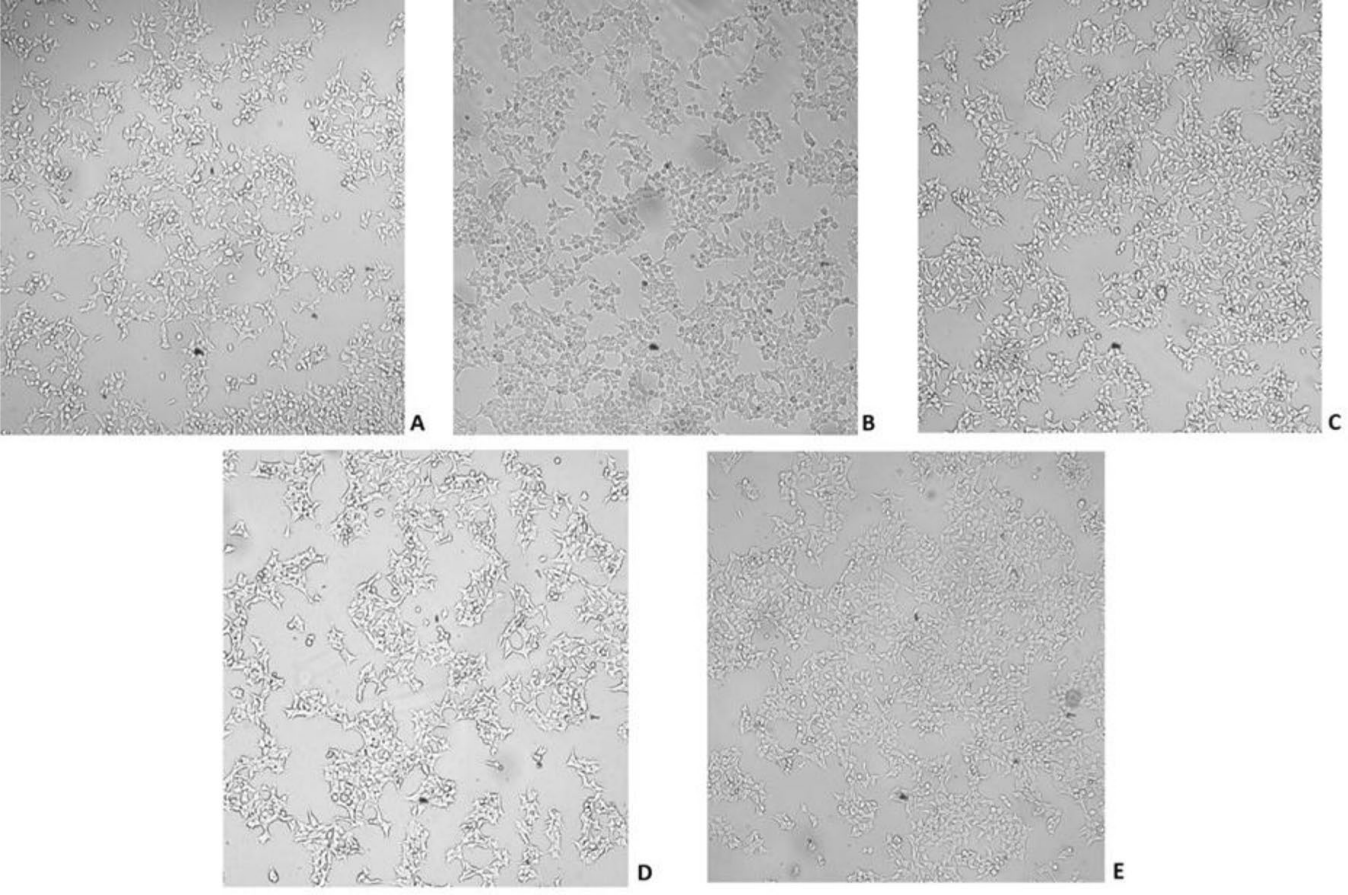
Images of HEK293T cell lines from a Nikon Eclipse TS2R microscope. (**A**) Control well. With added tested agents: (**B**) Propiophenone oxime, (**C**) β-ionone oxime, (**D**) (+)-carvone oxime, and E. norcamphor oxime. On comparison of the images, it can be stated that the tested compounds do not affect the morphology or viability of the cell line.

To evaluate the cytotoxic effects of the compounds on human cells, we performed a screening on the HEK293T cell line. Cells were treated with 25 µM of each tested oxime (and its corresponding carbonyl compound) for 24 h, and cell viability was determined using the MTS assay. In addition, we assessed alterations in cell morphology after treatments. No changes were observed in cells treated with tested compounds, compared to untreated cells (Fig. 1). Moreover, the percentage of cell viability calculated during the MTS assay confirms these observations (Table 2). None of the compounds exhibited toxic effects on HEK293T cells (Table S3), which supported the results of the ADME-Tox test.

**Table 2:** The percentage of viable HEK293T cells after 24 h of incubation with 25 µM of the indicated oxime. *SD* standard deviation.

Oxime of	Viability %	SD	Error %	Control (viability %)
Propiophenone	91.95	1.41	1.53	100.00
β-Ionone	100.00	5.30	5.30	100.00
(+)-Carvone	90.21	3.96	4.39	100.00
Norcamphor	93.46	4.61	4.94	100.00

### Evaluation of antimicrobial activity of oximes

The antimicrobial effects of low molecular weight carbonyl compounds and their corresponding oximes were assayed against a wide range of human and foodborne microorganisms, and their potency was assessed by evaluating the presence of inhibition zones. For every microorganism, at least one compound was identified that showed growth-inhibiting activity. Compared to the antibiotics used, many compounds did not exhibit comparable antimicrobial activity, but some of them showed MIC within the range of 100–1000 mg/L, which is routinely used for the classification of phytochemicals as antimicrobials^19^. The results of the screening disc diffusion assay allowed us to select active compounds for MIC evaluation (AlamarBlue ® cell viability assay). The only exception was *L. pneumophila.* Due to the difficulties in solid medium culture, screening tests were performed at one concentration (2400 mg/L) in liquid culture with Alamar blue® reagent to assess the viability of the bacteria. The MIC results were compared to well-known antimicrobial agents: ampicillin, gentamicin, ciprofloxacin, amphotericin B, fluconazole, and miconazole. The most relevant MIC values are shown in Table 3. Detail information on all tested compounds and strains tested can be found in supplementary data (Tables S1 and S2). As a result of the antimicrobial assay, *p*-anisaldehyde and its oxime, eucarvone and (+)-menthone, α-methyl-*trans*-cinnamaldehyde oxime, *o*-anisaldehyde oxime, β-cyclocitral oxime, isophorone, (+)-carvone, (+)-dihydrocarvone, methyl jasmonate oxime, and (±)-camphor showed no inhibitory activity against any of the tested strains. *Pseudomonas aeruginosa* was the most resistant bacteria and no compound showed any relevant inhibitory activity against it. α-Ionone oxime, β-ionone, β-ionone oxime, dihydro-α-ionone oxime, dihydro-β-ionone, dihydro-β-ionone oxime, β-cyclocitral, α-hexylcinnamaldehyde oxime exhibited relevant inhibitory activity against *L. pneumophila*. The best result obtained against these bacteria was by β-ionone oxime (37.50 mg/L, 0.18 mM). (−)-Camphor and its oxime were significantly active against *E. coli* (75.50 mg/L, 0.50 mM) and its oxime (150.00 mg/L, 0.90 mM).

**Table 3:** The most relevant MIC values.

Microorganism	Compound	MIC [mg/L] (mM)
*E .coli*	(−)-Camphor	75.50 (0.49)
	(−)-Camphor oxime	150.00 (0.90)
	Ampicillin	25.00 (0.07)^20^
	Gentamicin	20.00 (0.04)^20^
*L. pneumophila*	α-Ionone oxime	112.50 (0.54)
	β-Ionone	150.00 (0.78)
	β-Ionone oxime	37.50 (0.18)
	Dihydro-α-ionone oxime	150.00 (0.72)
	Dihydro-β-ionone	300.00 (1.54)
	Dihydro-β-ionone oxime	150.00 (0.72)
	β-Cyclocitral	300.00 (1.97)
	Gentamicin	0.39 (8.00 × 10^−4^)^21^
*E. hirae*	α-Amylcinnamaldehyde oxime	150.00 (0.74)
	α-Hexylcinnamaldehyde oxime	42.18 (0.18)
	*p*-Tolualdehyde oxime	150.00 (1.11)
	α-Isomethylionone oxime	18.75 (0.08)
	Ciprofloxacin	8.00 (0.02)^22^
*B. cereus*	Pseudoionone oxime	150.00 (0.72)
	Gentamicin	2.00 (4.00 × 10^−3^)^23^
	Ampicillin	1.00 (3.00 × 10^−3^)^23^
*S. aureus*	α-Hexylcinnamaldehyde	225.00 (1.04)
	α-Hexylcinnamaldehyde oxime	37.5. (0.16)
	Phenylacetaldehyde	42.18 (0.35)
	β-Ionone oxime	37.50 (0.18)
	Pseudoionone oxime	37.50 (0.18)
	Safranal oxime	150.00 (0.91)
	Geranyl acetone oxime	150.00 (0.72)
	Ampicillin	10.00 (0.03)^20^
	Gentamicin	5.00 (0.01)^20^
*A. brasiliensis*	*trans*-Cinnamaldehyde	112.50 (0.85)
	*trans*-Cinnamaldehyde oxime	37.50 (0.25)
	2-Phenylpropionaldehyde	37.50 (0.28)
	Piperonal	112.50 (0.74)
	(±)-Citronellal oxime	150.00 (0.89)
	*cis*-Jasmone	75.00 (0.45)
	*cis*-Jasmone oxime	37.50 (0.21)
	Dihydrocinnamaldehyde oxime	75.00 (0.50)
	Amphotericin B	1.00 (1.00 × 10^−3^)^24^
*C. albicans*	*trans*-Cinnamaldehyde	37.50 (0.28)
	*trans*-Cinnamaldehyde oxime	75.00 (0.51)
	Veratraldehyde	150.00 (0.90)
	Ethylvanillin	150.00 (0.90)
	Pseudoionone	112.50 (0.58)
	Pseudoionone oxime	37.50 (0.18)
	(−)-Carvone	112.50 (0.74)
	(−)-Carvone oxime	225.00 (1.36)
	Amphotericin B	1.00 (1.00 × 10^−3^)^23^
	Fluconazole	1.00 (3.00 × 10^−3^)^23^
	Miconazole	2.00 (5.00 × 10^−3^)^23^

Furthermore, α-hexylcinnamaldehyde, α-hexylcinnamaldehyde oxime, phenylacetaldehyde, β-ionone oxime, safranal oxime, geranyl acetone oxime exhibited activity against *S. aureus*. Moreover, pseudoionone oxime had relevant inhibitory activity against both *B. cereus* (150.00 mg/L, 0.72 mM) and *S. aureus* (37.50 mg/L, 0.18 mM). The best inhibitory activity against *S. aureus* was demonstrated by α-hexylcinnamaldehyde oxime (37.50 mg/L, 0.16 mM). α-Amylcinnamaldehyde oxime, α-hexylcinnamaldehyde oxime, *p*-tolualdehyde oxime showed antibacterial activity against *E. hirae* and the best result was obtained by α-isomethylionone oxime (18.75 mg/L, 0.08 mM). Among fungi, *trans*-cinnamaldehyde and its oxime were active against mold and yeast (MIC ranging from 37.50 (0.28) to 112.50 (0.85) mg/L(mM). In addition, 2-phenylpropionaldehyde, piperonal, (±)-citronellal oxime, *cis*-jasmone, dihydrocinnamaldehyde oxime, and *cis*-jasmone oxime exhibited antifungal activity against *A. brasiliensis*. On the contrary, veratraldehyde, ethylvanillin, pseudoionone, (−)-carvone, (−)-carvone oxime, and pseudoionone oxime showed growth inhibitory activity against *C. albicans,* the last was the most potent (37.50 mg/L, 0.18 mM).

## Discussion

**Figure 2:**
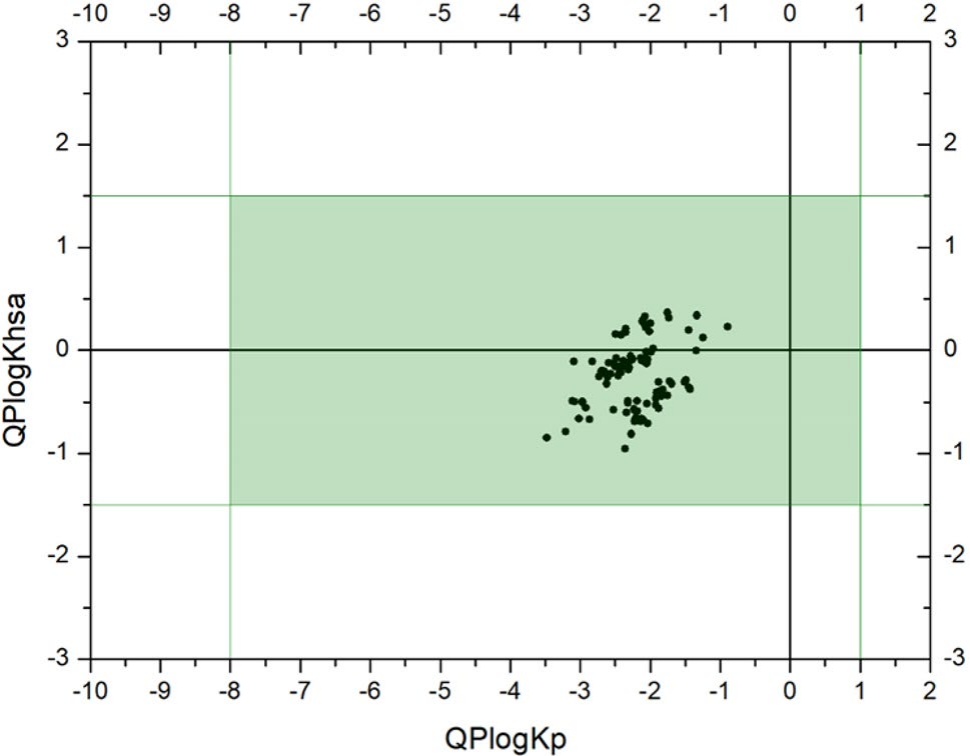
Graph showing the calculated parameters related to the biopermeability of compounds for humans: QPlogKp (predicted skin permeability, log Kp.), QPlogKhsa (prediction of binding to human serum albumin). The green area shows the normal range of values.

Only a few low-molecular compounds considered in this study were previously tested by ADME-Tox. Camphor, 1,8-cineole, dihydrocarvone, fenchone, piperitone, piperonal, myrtenal, benzaldehyde, cinnamaldehyde, citral, citronellal, vanillin, 2-phenylacetaldehyde, carvone, dihydrocinnamaldehyde, and geranylacetone were analyzed and fulfilled Lipinski’s rule of five^25^. For practically all carbonyl compounds, calculations were performed as well, but with the application of other tools^25–30^. Therefore, it was necessary to study compounds of interest with one algorithm to be able to compare computational results. In silico studies have shown that most parameters are within the acceptable range (according to QikPro 3.5 User Manual), indicating that our compounds of interest are potentially non-toxic antibacterial agents. The most important toxic properties, such as the predicted IC_50_ value for blockage of HERG K+ channels (QPlogHERG) and QPPCaco, do not show deviations. Since antimicrobials are often used as aerosols, it is important to determine their skin permeability (QPlogKP) and albumin binding profiles (QPlogKhsa) (Fig. 2). The results of these calculations were within the normal range.

The results of the in vitro MTS assay were consistent with the ADME-Tox results. All tested compounds were non-toxic to HEK293T cell lines (more than 85% survival rates). Furthermore, we did not detect any changes in cell morphology following treatments. When the results of both ADME-Tox and MTS assays are compared, it can be concluded that none of the compounds considered is toxic and might be considered safe to use in the environment inhabited by people.

The antimicrobial activity of low-molecular ingredients of essential oils and aromatic extracts was and is a subject of extensive antimicrobial evaluations; however, minor constituents of natural matrices such as oximes have been neglected, and almost no data can be found in the literature. The antimicrobial activities of naturally occurring compounds against pathogens can be evaluated with a broad number of assays. Each method has advantages and disadvantages, and in vitro results are difficult to compare due to the different methods, techniques, microorganisms, cells, and sources of antimicrobials used^31^. To obtain comparable results, it is important to perform the study with the same method, under the same conditions, and on the same strains of microorganisms. Therefore, despite the relatively abundant antimicrobial data for low-molecular carbonyl compounds, we had to reevaluate their activities to be able to compare them with our target compounds. It was also necessary to confirm the hypothesis that oximation leads to the enhancement of antimicrobial activity. In this study, universal tryptone broth was chosen for bacteria and fungi, which is a simple medium that supports good microbial growth. In our study, we focused on antimicrobial activity against common pathogens that inhabit HVAC systems in a home, hospital, and industrial environment. Among the tested low molecular weight compounds tested, several demonstrated satisfactory antimicrobial activity. In comparison to well-known antibiotics, all compounds were less potent towards considered microbial strains. Although, compared to other antimicrobials of natural origin, the antimicrobial potential is within the acceptable range. Many essential oils known for their antimicrobial activity possess lower MICs against species considered because they are compositions of low-molecular compounds and a synergistic action might be observed. Lavender oil used in urinary tract infections and burn treatment^32^ proved antimicrobial activity in a range of 1.25–5 mg/mL (*B. subtilis* ATCC 6633, *S. aureus* ATCC 25923 and *E. coli* ATCC 25922)^33^, clove essential oil used in dental products to prevent gum and teeth infections 1250–10,000 µL/mL (*S. aureus* (ATCC 6538), *P. aeruginosa* (ATCC 27853), *E. coli* (ATCC 8739)^34^. Some of the terpenoid constituents of essential oils that are commonly considered antiseptics showed lower antimicrobial activity such as eugenol 33.43–66.87 mg/mL (*S. aureus, B. cereus, E.coli*)^35^ used in dentistry^35,36^ or thymol 7.53–15.07 mg/mL (*S. aureus, B. cereus, E.coli*)^35^, which is a constituent of Listerine® mouthwash^37^.

In our study, we have confirmed that the oxime moiety might have an impact on antimicrobial activity. Low molecular weight oximes were, in general, more active than carbonyl compounds. Often, when the carbonyl compound did not have antimicrobial activity, its oxime showed moderate activity. In some cases, the oximes were significantly more active than the corresponding carbonyl compound. For example α-isomethyl ionone (Table S2, 1200.00 mg/L was 64-times weaker than its oxime against *E. hirae* (Table 3, 18.75 mg/L). Repeatedly, when the carbonyl compound exhibited antimicrobial activity, its oxime was also more active in most cases, but the difference was not significant. More compounds were active toward Gram-positive bacteria than against Gram-negative bacteria, and the same pattern can be observed for oximes. This might be related to the differences in cell structures—Gram-negative bacteria have a largely impermeable cell wall and outer lipid membrane, which might be less permeable to low-molecular compounds. More studies are needed to confirm this hypothesis.

Monoterpenoids are frequently evaluated natural carbonyl compounds for the antimicrobial activity. Camphor and its isomers are another monoterpenoids with antimicrobial potential. In the study by Kędzia et al. camphor (stereochemistry not specified by the authors) showed MIC values against *S.* *aureus, E.* *coli,* and *C.* *albicans* of 300 mg/L, 500 mg/L, and 400 mg/L respectively^38^. Our result indicates that the enantiomeric form of camphor has the influence on the antimicrobial activity and it is important to distinguish the results between isomers. (±)-Camphor showed no activity and (+)-camphor was slightly active against *S. aureus* (MIC = 1200.00 mg/L). (−)-Camphor was considerably active against *E. coli* (MIC = 75.00 mg/L) and slightly active against *E.* *hirae* (MIC = 1200.00 mg/L). Oximation of (−)-camphor resulted in lower anti-*E. coli* activity (MIC = 150.00 mg/L). Another carbonyl compound considered as an antimicrobial compound was fenchone. Literature studies showed that fenchone (stereochemistry not specified by the authors) has an antimicrobial potential (MIC value of 200 mg/L against *S. aureus* and *E. coli*)^38^*.* Surprisingly, in our study neither (−)-fenchone nor (+)-fenchone were active against these bacteria. However, (−)-fenchone was slightly active against *E.* *hirae* with MIC = 600.00 mg/L. Oximation of (−)-fenchone had no influence on the anti-*E. hirae* properties but it improved the antimicrobial properties of (+)-fenchone (MIC change from 2400 to 300 mg/L for ketone and oxime respectively).

Seven oximes exhibited some antifungal activity, which is a promising observation, as there are not many published indications of the activity of unsubstituted oximes against fungal species. Cinnamaldehyde occurs in nature predominately as a *trans*-isomer and was previously tested on various microorganisms, e.g.* E. coli*^39^, *P. aeruginosa*^40^. Regarding the antifungal activity of cinnamaldehyde against *C. albicans*, the reported values were 125 mg/L^41^ and 40 mg/L^42^. For *A. niger*, the reported MIC was 40 mg/L^43^, and the EC_50_ value reported by Marei and Abdelgalei was 3.19 mg/L^44^. The MIC values in our study against *A. brasiliensis* and *C. albicans* were 37.50 mg/L and 112.50 mg/L, respectively. Compared to other cinnamaldehyde derivatives, *trans-*cinnamaldehyde had the broadest spectrum of activity. Neither *trans*-cinnamaldehyde nor its oxime had the activity against gram-positive bacteria, while α-hexylcinnamaldehyde oxime showed good inhibitory activity against *E.* *hirae* and *S. aureus*. Similar MIC values against fungal species can be attributed to hydrocinnamaldehyde oxime and 2-phenylpropionaldehyde. According to our studies, *cis*-jasmone has proven to be potent against *A.* *brasiliensis* (MIC = 75.00 mg/L), and not active against *C. albicans*. Oxime of *cis*-jasmone was more active against both fungal species (MIC = 37.5 and 300 mg/L for *A.* *brasiliensis* and *C. albicans* respectively). Taking into account the results available in the literature^45,46^ and the results obtained in our study, *cis*-jasmone and its oxime might be considered a universal substance that satisfactory inhibits the growth of both bacteria and yeast. Unfortunately, when focusing on the results of the strains associated with HVAC systems, this substance cannot be considered as a sufficient component for the functional perfumery.

Among the benzaldehyde derivatives, ethyl vanillin and its oxime were previously investigated for their antimicrobial potential. In the study conducted by Morris et al. ethyl vanillin proved moderate activity toward *E.coli* (MIC = 1000 ppm) and *C. albicans* (MIC = 1000 ppm)^47^, while in other studies its oxime did not prove growth inhibitory activity against *S. aureus* (MIC > 50,000 mg/L) and *P. aeruginosa* (MIC > 50,000 g/L)^48^. In our study ethylvanillin presented very good activity towards *C. albicans* (MIC = 150.00 mg/L) and moderate activity toward *A. brasiliensis* (MIC = 300.00 mg/L) and *L. pneumophila* (MIC = 300.00 mg/L) while its oxime weakly inhibited growth of *E. hirae* (MIC = 600.00 mg/L)*, C. albicans* (MIC = 900.00 mg/L) and moderately of *A. brasiliensis* (MIC = 300.00 mg/L)*.* On the basis of our results, ethyl vanillin is an antifungal agent with possible application in household chemicals.

In conclusion, low-molecular oximes might be considered as potential supporting antimicrobial agents. Computational studies predicted the significant potential of those compounds as therapeutic substances, and their low toxicity potential. The low toxicity profile was confirmed in studies on the human HEK293T cell line. Moreover, ADME-tox confirmed the potential safety of these substances when used as aerosols. Oximation of naturally occurring carbonyl compounds seems to be a good direction for the enhancement of antimicrobial activity. In this respect, it can be concluded that some of the evaluated oximes but also some of the carbonyl compounds can be used as facilitators of inhibition of the development of microorganisms in household chemicals and cosmetics.

## Methods

### Low molecular carbonyl compounds

Almost all compounds were obtained from Merck Poland, except *trans*-cinnamaldehyde, α-hexylcinnamaldehyde, *p*-tolualdehyde, piperitone (Tokyo Chemical Industry Co. LTD), piperonal (LOBA Chemie Austria), and vanillin (Avantor Performance Materials Poland S.A). All corresponding oximes were synthesized in our laboratory and are described in our previous communication^10^.

### In-silico ADME-Tox

Seventy-nine compounds were used for in silico studies. All of them were optimized and neutralized using an OPLSe force field. The QikProp tool^49^ calculated the ADME and molecular properties of inhibitors with identification to the five most similar drugs. The QikProp algorithm uses the implemented database of the compounds. The compounds are described by their ADMET predicted properties verified by the experimental data. The set of the over 1700 drugs is compared with the novel chemical compounds during the calculations. The key properties solubility (logS), permeability (logP), similarities in molecular property space are used for the selection of the best score. The data file can be modified and enlarged by the additional molecules from different types of drugs (natural-like, peptides, synthetic, etc.). Detailed data on the properties analyzed are included in the supporting materials.

### MTS assay

This study was carried out using HEK293T cell lines from ATCC license distributor (LGC Standards Sp. z o. o., ATCC® CRL-3216). HEK293T cells were cultured in Dulbecco’s Modified Eagle’s Medium (DMEM) (Corning Cellgro) supplemented with 10% Fetal Bovine Serum (heat inactivated) (FBS, Atlas Biologicals), 2 mM L-glutamine (Omega Scientific), 100units/mL penicillin and 10 ug/mL streptomycin (both Gibco Life Technologies) in a humidified 5% CO_2_ atmosphere at 37 °C. MTS was obtained from Promega (CellTiter 96® AQueous One Solution Cell Proliferation Assay). Cells were seeded in 96-well cell culture plates (20,000 cells per well in 100 μL of phenol red-free DMEM medium supplemented with 10% FBS, 2 mM l-glutamine, 100 units/mL penicillin and 10 ug/mL streptomycin) and allowed to attach overnight. The next day, the medium was changed and the cells were treated with test compounds (25 μM) for 24 h. After this time, cell viability was determined using an MTS assay according to the manufacturer’s protocol (CellTiter 96® Aqueous One Solution Cell Proliferation Assay, G3582, Promega). The number of living cells is reported as a survival ratio calculated based on the absorbance value measured in control wells (untreated cells). All measurements were performed in triplicate. Images of cells before and after treatment were acquired with a fluorescence microscope Nikon Eclipse TS2R (objective 10x, Differential Interference Contrast mode). Cell morphology was observed using 10× or 40× objective.

### Antimicrobial activity

The microorganisms used in this study represent pathogenic species commonly associated with healthcare and household environments. Microorganisms were obtained from MediMark Europe and TCS Biosciences Ltd. and consisted of the Gram-positive strains *Enterococcus hirae* (ATCC 10541) (MediMark), *Bacillus cereus* (ATCC 10876) (TCS), and *Staphylococcus aureus* (ATCC 6538) (MediMark); the Gram-negative strains *Escherichia coli* (ATCC 10536) (MediMark), *Pseudomonas aeruginosa* (ATCC 15442) (TCS), and *Legionella pneumophila* (ATCC 33152) (TCS); and the fungi *Aspergillus brasiliensis* (ATCC 16404) (MediMark), and *Candida albicans* (ATCC 10231) (MediMark). Bacteria and fungi were maintained in the Department of Chemical Biology and Bioimaging, Wroclaw University of Science and Technology. All bacterial strains were subcultured from the original culture and maintained on Nutrient LAB-AGAR™ (Biocorp Poland) plates at 4 °C and grown at 37 °C when required. All fungal strains were subcultured from the original culture and kept in Sabouraud Dextrose LAB-AGAR™ (Biocorp Poland) plates at 4 °C and grown at 28 °C when required. The Alamarblue® reagent was obtained from Bio-Rad-Antibodies UK. Microplates were obtained from NEST Biotechnology Co., Ltd. China.

### Antibacterial activity screening-disc diffusion assay

Antibacterial effects were preliminarily screened using a paper disc diffusion method. All bacterial strains were subcultured in Nutrient Broth (Biocorp) and incubated at 37 °C for 24 h, and then bacterial cells were suspended in saline solution according to McFarland’s protocol^50^ to produce suspensions of about 1–2 × 10^8^ CFU mL^−1^. The agar plates were then impregnated with 250 µL of this suspension and spread on agar. The discs (6 mm in diameter) were impregnated with 10 µL diluted in methanol to a final concentration of 30 mg/mL of test agent (300 µg/disc) placed on the inoculated agar. Pure methanol (10 µL) was used as a negative control, while ofloxacin discs (5 µg) and netilmicin discs (30 µg) were used as positive reference standards to determine the sensitivity of each strain of bacterial species tested. Plates were observed after 24 h at 37 °C. All tests were carried out in triplicate, and antibacterial activity is expressed as the mean of inhibitory zones (mm) produced by each test agent.

### Antifungal activity screening: disc diffusion assay

Antifungal effects were tested using a paper disc diffusion method. Overnight broth (Sabouraud Dextrose Broth—Biocorp) yeast culture was adjusted to 1 × 10^7^ CFU mL^−1^ according to McFarland’s protocol. For mold, suspension of mature spores was obtained by gently washing the surface of the solid medium with a 0.05% (v/v) solution of Tween 80 (Greenaction), and the resulting suspension was adjusted to 10^6^ spores mL^−1^. Then, agar plates were impregnated with 250 µL of stock suspensions, yeast and mold were spread on their respective agar plates. The discs (6 mm diameter) were impregnated with 10 µL (30 mg/mL) of test agent (300 µg/disc) placed on inoculated agar. Pure methanol (10 µL) was used as a negative control, while fluconazole discs (25 µg) for yeast and netilmicin discs (30 µg) for mold were used as positive reference standards to determine the sensitivity of each strain of bacterial species tested. Plates were observed after 48 and 72 h at 28 °C. All tests were performed in triplicate, and antifungal activity is expressed as the mean of the inhibitory zones (mm) produced by each test agent.

### Analysis of disc diffusion assays

To present comparable results, a consistent analysis was performed. The methanol negative control did not show inhibitory effects. Positive controls showed inhibition diameters ranging from 12 to 30 mm (ofloxacin), 25–28 mm (netilmicin), and 22–25 mm (fluconazole). All inhibition zones were compared with the results for antibiotics for all microorganisms. If the inhibition zone was smaller than 10 mm, it was considered that was no activity, if smaller than 13 mm was considered medium active and higher than 13 mm strong activity based on the results of positive controls, which were considered maximal inhibitory zones.

### Microwell dilution assay

Minimal inhibitory concentration (MIC) values were determined for all microbial strains sensitive to the carbonyl compound and/or oxime in disc diffusion assay. The test was carried out in 96-well plates using the Alamarblue® viability assay protocol in quadruplicate.

### MIC evaluation on bacterial strains

All bacterial strains were subcultured in Nutrient Broth (Biocorp) and incubated at 37 °C for 24 h, and then the turbidity of the inoculum was adjusted to obtain concentrations of 5 × 10^5^ CFU mL^−1^ (OD_550_ = 0.125). All compounds tested were diluted to 10 different concentrations, starting with 4.8 mg/mL and serial doubling dilutions were performed in 96-well microplates (NEST). Dimethylsulfoxide (DMSO) solution (67.2 µL), Nutrient Broth (Biocorp) (67.2 µL), and 25.6 µL of the previously prepared test agent were added to the wells of column 1, while the remaining wells 2–10 received 80 µL of Nutrient Broth (Biocorp). Two-fold serial dilutions were prepared horizontally on the plate. Excess dilutions (80 µL) were discarded from column 10. Four replicates were made for each test agent. Columns 11 and 12 constituted controls to which 40 µL of Nutrient Broth (Biocorp) and 40 µL of distilled sterile water were added (column 11) to which 90 µL of Nutrient Broth (Biocorp) and 10 µL of distilled sterile water were added (column 12). To the wells for columns 1–11, 10 µL of liquid culture were added. The plate was incubated for two hours at 37 °C, and then a 10 µL aliquot of AlamarBlue ® color reagent was added to each well. Plates were incubated for 24 h at 37 °C and then analyzed.

### MIC evaluation on fungal strains

Mold strains were subcultured in Sabouraud Dextrose LAB-AGAR™ and incubated at 28 °C for 72 h. The suspension of mature spores was obtained by gently washing the surface of solid media with a 0.05% (v/v) solution of Tween 80 (Greenaction), and the resulting suspension was adjusted to 2.5 × 10^4^ spores mL^−1^. For yeast, overnight broth culture (Sabouraud Dextrose Broth—Biocorp) was adjusted to 1 × 10^6^ CFU mL^−1^ according to McFarland’s protocol. Plates were prepared analogously to the MIC evaluation for bacterial strains, although instead of Nutrient Broth (Biocorp), Sabouraud Dextrose Broth (Biocorp) was used. The plates were incubated for two hours at 28 °C, and then a 10 µL aliquot of AlamarBlue ® color reagent was added to each well. The plates were incubated for an additional 48 h (yeast) or 72 h (mold) at 28 °C and then analyzed.

### Analysis of microwell dilution assays

The color change from blue to red was taken as an indication of bacterial growth as described by Baker et al.^51^. MIC was determined as the concentration of the compound (μg per mL of medium) in the first well where the mixture remained blue. MIC was considered the lowest concentration that resulted in a significant decrease in inoculum viability.

## Supplementary Information

Supplementary Information 1.

Supplementary Information 2.

## Data Availability

All data generated or analyzed during this study are included in this published article.
