# Supplementary material for: Cytotoxicity, early safety screening, and antimicrobial potential of minor oxime constituents of essential oils and aromatic extracts
Source: Sci Rep. 2022 Mar 29;12:5319. doi: 10.1038/s41598-022-09210-z (PMC8964709; doi:10.1038/s41598-022-09210-z)
Supplement: Supplementary file 1 — Supplementary Information 1. [file 41598_2022_9210_MOESM1_ESM.docx]

**Supplementary Information**

Supporting figures and tables (Disc diffusion assay, MIC, Lipinski's rule of five properties, SASA chart, Predicted polarizability, solubility, and partition coefficients, QPPCaco, QPlogBB, QPPMDCK, QPlogBB chart with min and max recommended values marked, APlog Kp, QPlogKhsa, Percent Human Oral Absorption, #Stars, MTS assay).
