# Supplementary material for: Cytotoxicity, early safety screening, and antimicrobial potential of minor oxime constituents of essential oils and aromatic extracts
Source: Sci Rep. 2022 Mar 29;12:5319. doi: 10.1038/s41598-022-09210-z (PMC8964709; doi:10.1038/s41598-022-09210-z)
Supplement: Supplementary file 2 — Supplementary Information 2. [file 41598_2022_9210_MOESM2_ESM.docx]

**Supporting information**

**Cytotoxicity, early safety screening, and antimicrobial potential of minor oxime constituents of essential oils and aromatic extracts**

Alicja K. Surowiak ^a^, Marta Sowała ^a^, Michał Talma ^b^, Katarzyna Groborz ^a^, Lucyna Balcerzak ^a^, Stanisław Lochyński ^a,c^, Daniel J. Strub ^a,d^*

^a^ Department of Chemical Biology and Bioimaging, Faculty of Chemistry, Wrocław University of Science and Technology, Wyb. Wyspiańskiego 27, 50-370 Wrocław, Poland

^b^ Department of Bioorganic Chemistry, Faculty of Chemistry, Wrocław University of Science and Technology, Wyb. Wyspiańskiego 27, 50-370 Wrocław, Poland

^c^ Institute of Cosmetology, Wroclaw College of Physiotherapy, T. Kościuszki 4, 50-038 Wrocław

^d^ Liquid Technologies sp. z o.o., Gdańska 13, 50-344 Wrocław

^*^ Corresponding author: Daniel Jan Strub, Department of Chemical Biology and Bioimaging, Faculty of Chemistry, Wrocław University of Science and Technology, Wyb. Wyspiańskiego 27, 50-370 Wrocław, Poland, telephone: +48 71 320 20 10, e-mail: [daniel.strub@pwr.edu.pl](mailto:daniel.strub@pwr.edu.pl).

**Table of contents**

[**Tab.S1** Results of screening test – Disc diffusion assay of low-molecular carbonyl compounds and its oxime 2](#_Toc69802243)

[**Tab.S2** Minimal Inhibitory Concentration of low-molecular carbonyl compounds and its oxime derivatives 14](#_Toc69802244)

[**Tab.S3** The Reasults MTS Assay For all tested low-molecular carbonyl compounds and its oxime derivatives 18](#_Toc69802245)

[**Tab.S4** The Results Table of SELECTED ADME-Tox properties for selected COMPOUNDS – PART I. 21](#_Toc69802246)

[**Tab.S5** The Results Table of SELECTED ADME-Tox properties for selected COMPOUNDS – PART II. 25](#_Toc69802247)

# TabLE S1. Results of screening test – Disc diffusion assay of low-molecular carbonyl compounds and THEIR oximeS

| **No** | **Compound** | | **Microorganism** | **Zone of inhibition [mm]** |
| --- | --- | --- | --- | --- |
| 1 | α-Amylcinnamaldehyde | | *E. coli* | 7 |
|  |  |  | *E. hirae* | 7 |
|  |  |  | *P. aeruginosa* | 7 |
|  |  |  | *B. cereus* | 8 |
|  |  |  | *S. aureus* | 10 |
|  |  |  | *A. brasiliensis* | 7 |
|  |  |  | *C. albicans* | 7 |
| 2 | α-Amylcinnamaldehyde oxime | | *E. coli* | 7 |
|  |  |  | *E. hirae* | 13 |
|  |  |  | *P. aeruginosa* | 7 |
|  |  |  | *B. cereus* | 7 |
|  |  |  | *S. aureus* | 8 |
|  |  |  | *A. brasiliensis* | 7 |
|  |  |  | *C. albicans* | 13 |
| 3 | *m*-Anisaldehyde | | *E. coli* | 7 |
|  |  |  | *E. hirae* | 10 |
|  |  |  | *P. aeruginosa* | 7 |
|  |  |  | *B. cereus* | 7 |
|  |  |  | *S. aureus* | 9 |
|  |  |  | *A. brasiliensis* | 7 |
|  |  |  | *C. albicans* | 7 |
| 4 | *m-*Anisaldehyde oxime | | *E. coli* | 9 |
|  |  |  | *E. hirae* | 7 |
|  |  |  | *P. aeruginosa* | 7 |
|  |  |  | *B. cereus* | 7 |
|  |  |  | *S. aureus* | 43 |
|  |  |  | *A. brasiliensis* | 7 |
|  |  |  | *C. albicans* | 7 |
| 5 | *o-*Anisaldehyde | | *E. coli* | 7 |
|  |  |  | *E. hirae* | 9 |
|  |  |  | *P. aeruginosa* | 8 |
|  |  |  | *B. cereus* | 8 |
|  |  |  | *S. aureus* | 11 |
|  |  |  | *A. brasiliensis* | 9 |
|  |  |  | *C. albicans* | 7 |
| 6 | *o-*Anisaldehyde oxime | | *E. coli* | 10 |
|  |  |  | *E. hirae* | 10 |
|  |  |  | *P. aeruginosa* | 7 |
|  |  |  | *B. cereus* | 7 |
|  |  |  | *S. aureus* | 7 |
|  |  |  | *A. brasiliensis* | 7 |
|  |  |  | *C. albicans* | 10 |
| 7 | *p*-Anisaldehyde | | *E. coli* | 7 |
|  |  |  | *E. hirae* | 7 |
|  |  |  | *P. aeruginosa* | 8 |
|  |  |  | *B. cereus* | 8 |
|  |  |  | *S. aureus* | 9 |
|  |  |  | *A. brasiliensis* | 10 |
|  |  |  | *C. albicans* | 7 |
| 8 | *p*-Anisaldehyde oxime | | *E. coli* | 9 |
|  |  |  | *E. hirae* | 7 |
|  |  |  | *P. aeruginosa* | 8 |
|  |  |  | *B. cereus* | 7 |
|  |  |  | *S. aureus* | 7 |
|  |  |  | *A. brasiliensis* | 10 |
|  |  |  | *C. albicans* | 7 |
| 9 | Benzaldehyde | | *E. coli* | 7 |
|  |  |  | *E. hirae* | 7 |
|  |  |  | *P. aeruginosa* | 8 |
|  |  |  | *B. cereus* | 7 |
|  |  |  | *S. aureus* | 10 |
|  |  |  | *A. brasiliensis* | 7 |
|  |  |  | *C. albicans* | 7 |
| 10 | Benzaldehyde oxime | | *E. coli* | 11 |
|  |  |  | *E. hirae* | 11 |
|  |  |  | *P. aeruginosa* | 7 |
|  |  |  | *B. cereus* | 7 |
|  |  |  | *S. aureus* | 44 |
|  |  |  | *A. brasiliensis* | 7 |
|  |  |  | *C. albicans* | 11 |
| 11 | (+)-Camphor | | *E. coli* | 7 |
|  |  |  | *E. hirae* | 10 |
|  |  |  | *P. aeruginosa* | 7 |
|  |  |  | *B. cereus* | 8 |
|  |  |  | *S. aureus* | 12 |
|  |  |  | *A. brasiliensis* | 7 |
|  |  |  | *C. albicans* | 8 |
| 12 | (+)-Camphor oxime | | *E. coli* | 10 |
|  |  |  | *E. hirae* | 7 |
|  |  |  | *P. aeruginosa* | 7 |
|  |  |  | *B. cereus* | 7 |
|  |  |  | *S. aureus* | 10 |
|  |  |  | *A. brasiliensis* | 7 |
|  |  |  | *C. albicans* | 7 |
| 13 | ( ̶ )-Camphor | | *E. coli* | 11 |
|  |  |  | *E. hirae* | 10 |
|  |  |  | *P. aeruginosa* | 7 |
|  |  |  | *B. cereus* | 9 |
|  |  |  | *S. aureus* | 9 |
|  |  |  | *A. brasiliensis* | 7 |
|  |  |  | *C. albicans* | 10 |
| 14 | ( ̶ )-Camphor oxime | | *E. coli* | 10 |
|  |  |  | *E. hirae* | 12 |
|  |  |  | *P. aeruginosa* | 7 |
|  |  |  | *B. cereus* | 7 |
|  |  |  | *S. aureus* | 7 |
|  |  |  | *A. brasiliensis* | 7 |
|  |  |  | *C. albicans* | 7 |
| 15 | (±)-Camphor | | *E. coli* | 7 |
|  |  |  | *E. hirae* | 11 |
|  |  |  | *P. aeruginosa* | 7 |
|  |  |  | *B. cereus* | 7 |
|  |  |  | *S. aureus* | 9 |
|  |  |  | *A. brasiliensis* | 8 |
|  |  |  | *C. albicans* | 8 |
| 16 | (±)-Camphor oxime | | *E. coli* | 9 |
|  |  |  | *E. hirae* | 7 |
|  |  |  | *P. aeruginosa* | 7 |
|  |  |  | *B. cereus* | 7 |
|  |  |  | *S. aureus* | 7 |
|  |  |  | *A. brasiliensis* | 7 |
|  |  |  | *C. albicans* | 14 |
| 17 | (+)-Carvone | | *E. coli* | 7 |
|  |  |  | *E. hirae* | 7 |
|  |  |  | *P. aeruginosa* | 7 |
|  |  |  | *B. cereus* | 8 |
|  |  |  | *S. aureus* | 10 |
|  |  |  | *A. brasiliensis* | 7 |
|  |  |  | *C. albicans* | 7 |
| 18 | (+)-Carvone oxime | | *E. coli* | 7 |
|  |  |  | *E. hirae* | 12 |
|  |  |  | *P. aeruginosa* | 7 |
|  |  |  | *B. cereus* | 7 |
|  |  |  | *S. aureus* | 7 |
|  |  |  | *A. brasiliensis* | 10 |
|  |  |  | *C. albicans* | 9 |
| 19 | ( ̶ )-Carvone | | *E. coli* | 7 |
|  |  |  | *E. hirae* | 7 |
|  |  |  | *P. aeruginosa* | 7 |
|  |  |  | *B. cereus* | 9 |
|  |  |  | *S. aureus* | 11 |
|  |  |  | *A. brasiliensis* | 7 |
|  |  |  | *C. albicans* | 17 |
| 20 | ( ̶ )-Carvone oxime | | *E. coli* | 7 |
|  |  |  | *E. hirae* | 11 |
|  |  |  | *P. aeruginosa* | 7 |
|  |  |  | *B. cereus* | 7 |
|  |  |  | *S. aureus* | 7 |
|  |  |  | *A. brasiliensis* | 9 |
|  |  |  | *C. albicans* | 11 |
| 21 | 1,8-Cineole | | *E. coli* | 7 |
|  |  |  | *E. hirae* | 12 |
|  |  |  | *P. aeruginosa* | 7 |
|  |  |  | *B. cereus* | 7 |
|  |  |  | *S. aureus* | 8 |
|  |  |  | *A. brasiliensis* | 8 |
|  |  |  | *C. albicans* | 7 |
| 22 | Oxo-1,8-cineole oxime | | *E. coli* | 10 |
|  |  |  | *E. hirae* | 10 |
|  |  |  | *P. aeruginosa* | 7 |
|  |  |  | *B. cereus* | 7 |
|  |  |  | *S. aureus* | 7 |
|  |  |  | *A. brasiliensis* | 7 |
|  |  |  | *C. albicans* | 7 |
| 23 | *trans-*Cinnamaldehyde | | *E. coli* | 12 |
|  |  |  | *E. hirae* | 8 |
|  |  |  | *P. aeruginosa* | 9 |
|  |  |  | *B. cereus* | 19 |
|  |  |  | *S. aureus* | 7 |
|  |  |  | *A. brasiliensis* | 23 |
|  |  |  | *C. albicans* | 23 |
| 24 | *trans-*Cinnamaldehyde oxime | | *E. coli* | 11 |
|  |  |  | *E. hirae* | 12 |
|  |  |  | *P. aeruginosa* | 7 |
|  |  |  | *B. cereus* | 7 |
|  |  |  | *S. aureus* | 7 |
|  |  |  | *A. brasiliensis* | 22 |
|  |  |  | *C. albicans* | 12 |
| 25 | Citral | | *E. coli* | 7 |
|  |  |  | *E. hirae* | 7 |
|  |  |  | *P. aeruginosa* | 13 |
|  |  |  | *B. cereus* | 13 |
|  |  |  | *S. aureus* | 11 |
|  |  |  | *A. brasiliensis* | 8 |
|  |  |  | *C. albicans* | 7 |
| 26 | Citral oxime | | *E. coli* | 8 |
|  |  |  | *E. hirae* | 7 |
|  |  |  | *P. aeruginosa* | 7 |
|  |  |  | *B. cereus* | 7 |
|  |  |  | *S. aureus* | 7 |
|  |  |  | *A. brasiliensis* | 7 |
|  |  |  | *C. albicans* | 7 |
| 27 | (±)-Citronellal | | *E. coli* | 7 |
|  |  |  | *E. hirae* | 12 |
|  |  |  | *P. aeruginosa* | 7 |
|  |  |  | *B. cereus* | 7 |
|  |  |  | *S. aureus* | 10 |
|  |  |  | *A. brasiliensis* | 7 |
|  |  |  | *C. albicans* | 9 |
| 28 | (±)-Citronellal oxime | | *E. coli* | 10 |
|  |  |  | *E. hirae* | 10 |
|  |  |  | *P. aeruginosa* | 8 |
|  |  |  | *B. cereus* | 9 |
|  |  |  | *S. aureus* | 13 |
|  |  |  | *A. brasiliensis* | 19 |
|  |  |  | *C. albicans* | 10 |
| 29 | β-Cyclocitral | | *E. coli* | 7 |
|  |  |  | *E. hirae* | 12 |
|  |  |  | *P. aeruginosa* | 13 |
|  |  |  | *B. cereus* | 10 |
|  |  |  | *S. aureus* | 9 |
|  |  |  | *A. brasiliensis* | 8 |
|  |  |  | *C. albicans* | 7 |
| 30 | β-Cyclocitral oxime | | *E. coli* | 10 |
|  |  |  | *E. hirae* | 10 |
|  |  |  | *P. aeruginosa* | 7 |
|  |  |  | *B. cereus* | 7 |
|  |  |  | *S. aureus* | 7 |
|  |  |  | *A. brasiliensis* | 7 |
|  |  |  | *C. albicans* | 10 |
| 31 | (+)-Dihydrocarvone | | *E. coli* | 7 |
|  |  |  | *E. hirae* | 9 |
|  |  |  | *P. aeruginosa* | 7 |
|  |  |  | *B. cereus* | 8 |
|  |  |  | *S. aureus* | 9 |
|  |  |  | *A. brasiliensis* | 7 |
|  |  |  | *C. albicans* | 7 |
| 32 | (+)-Dihydrocarvone oxime | | *E. coli* | 9 |
|  |  |  | *E. hirae* | 10 |
|  |  |  | *P. aeruginosa* | 7 |
|  |  |  | *B. cereus* | 7 |
|  |  |  | *S. aureus* | 7 |
|  |  |  | *A. brasiliensis* | 7 |
|  |  |  | *C. albicans* | 10 |
| 33 | Dihydrocinnamaldehyde | | *E. coli* | 7 |
|  |  |  | *E. hirae* | 7 |
|  |  |  | *P. aeruginosa* | 8 |
|  |  |  | *B. cereus* | 13 |
|  |  |  | *S. aureus* | 10 |
|  |  |  | *A. brasiliensis* | 7 |
|  |  |  | *C. albicans* | 7 |
| 34 | Dihydrocinnamaldehyde oxime | | *E. coli* | 7 |
|  |  |  | *E. hirae* | 13 |
|  |  |  | *P. aeruginosa* | 7 |
|  |  |  | *B. cereus* | 7 |
|  |  |  | *S. aureus* | 7 |
|  |  |  | *A. brasiliensis* | 7 |
|  |  |  | *C. albicans* | 13 |
| 35 | (±)-Dihydrocitronellal | | *E. coli* | 11 |
|  |  |  | *E. hirae* | 7 |
|  |  |  | *P. aeruginosa* | 7 |
|  |  |  | *B. cereus* | 10 |
|  |  |  | *S. aureus* | 10 |
|  |  |  | *A. brasiliensis* | 7 |
|  |  |  | *C. albicans* | 10 |
| 36 | (±)-Dihydrocitronellal oxime | | *E. coli* | 10 |
|  |  |  | *E. hirae* | 11 |
|  |  |  | *P. aeruginosa* | 7 |
|  |  |  | *B. cereus* | 10 |
|  |  |  | *S. aureus* | 9 |
|  |  |  | *A. brasiliensis* | 9 |
|  |  |  | *C. albicans* | 11 |
| 37 | Dihydrojasmone | | *E. coli* | 8 |
|  |  |  | *E. hirae* | 8 |
|  |  |  | *P. aeruginosa* | 7 |
|  |  |  | *B. cereus* | 10 |
|  |  |  | *S. aureus* | 13 |
|  |  |  | *A. brasiliensis* | 9 |
|  |  |  | *C. albicans* | 9 |
| 38 | Dihydrojasmone oxime | | *E. coli* | 9 |
|  |  |  | *E. hirae* | 12 |
|  |  |  | *P. aeruginosa* | 7 |
|  |  |  | *B. cereus* | 7 |
|  |  |  | *S. aureus* | 22 |
|  |  |  | *A. brasiliensis* | 7 |
|  |  |  | *C. albicans* | 10 |
| 39 | Dihydro-α-ionone oxime | | *E. coli* | 15 |
|  |  |  | *E. hirae* | 7 |
|  |  |  | *P. aeruginosa* | 8 |
|  |  |  | *B. cereus* | 7 |
|  |  |  | *S. aureus* | 7 |
|  |  |  | *A. brasiliensis* | 7 |
|  |  |  | *C. albicans* | 7 |
| 40 | Dihydro-β-ionone | | *E. coli* | 7 |
|  |  |  | *E. hirae* | 7 |
|  |  |  | *P. aeruginosa* | 7 |
|  |  |  | *B. cereus* | 11 |
|  |  |  | *S. aureus* | 10 |
|  |  |  | *A. brasiliensis* | 7 |
|  |  |  | *C. albicans* | 7 |
| 41 | Dihydro-β-ionone oxime | | *E. coli* | 7 |
|  |  |  | *E. hirae* | 12 |
|  |  |  | *P. aeruginosa* | 7 |
|  |  |  | *B. cereus* | 7 |
|  |  |  | *S. aureus* | 7 |
|  |  |  | *A. brasiliensis* | 7 |
|  |  |  | *C. albicans* | 12 |
| 42 | Ethylvanillin | | *E. coli* | 7 |
|  |  |  | *E. hirae* | 7 |
|  |  |  | *P. aeruginosa* | 7 |
|  |  |  | *B. cereus* | 9 |
|  |  |  | *S. aureus* | 9 |
|  |  |  | *A. brasiliensis* | 15 |
|  |  |  | *C. albicans* | 12 |
| 43 | Ethylvanillin oxime | | *E. coli* | 7 |
|  |  |  | *E. hirae* | 12 |
|  |  |  | *P. aeruginosa* | 7 |
|  |  |  | *B. cereus* | 7 |
|  |  |  | *S. aureus* | 7 |
|  |  |  | *A. brasiliensis* | 7 |
|  |  |  | *C. albicans* | 12 |
| 44 | Eucarvone oxime | | *E. coli* | 8 |
|  |  |  | *E. hirae* | 9 |
|  |  |  | *P. aeruginosa* | 7 |
|  |  |  | *B. cereus* | 7 |
|  |  |  | *S. aureus* | 7 |
|  |  |  | *A. brasiliensis* | 7 |
|  |  |  | *C. albicans* | 10 |
| 45 | (+)-Fenchone | | *E. coli* | 7 |
|  |  |  | *E. hirae* | 11 |
|  |  |  | *P. aeruginosa* | 7 |
|  |  |  | *B. cereus* | 7 |
|  |  |  | *S. aureus* | 7 |
|  |  |  | *A. brasiliensis* | 7 |
|  |  |  | *C. albicans* | 8 |
| 46 | (+)-Fenchone oxime | | *E. coli* | 10 |
|  |  |  | *E. hirae* | 14 |
|  |  |  | *P. aeruginosa* | 7 |
|  |  |  | *B. cereus* | 7 |
|  |  |  | *S. aureus* | 7 |
|  |  |  | *A. brasiliensis* | 7 |
|  |  |  | *C. albicans* | 7 |
| 47 | ( ̶ )-Fenchone | | *E. coli* | 7 |
|  |  |  | *E. hirae* | 14 |
|  |  |  | *P. aeruginosa* | 7 |
|  |  |  | *B. cereus* | 7 |
|  |  |  | *S. aureus* | 7 |
|  |  |  | *A. brasiliensis* | 7 |
|  |  |  | *C. albicans* | 8 |
| 48 | ( ̶ )-Fenchone oxime | | *E. coli* | 8 |
|  |  |  | *E. hirae* | 14 |
|  |  |  | *P. aeruginosa* | 7 |
|  |  |  | *B. cereus* | 7 |
|  |  |  | *S. aureus* | 7 |
|  |  |  | *A. brasiliensis* | 7 |
|  |  |  | *C. albicans* | 8 |
| 49 | Geranylacetone | | *E. coli* | 7 |
|  |  |  | *E. hirae* | 8 |
|  |  |  | *P. aeruginosa* | 7 |
|  |  |  | *B. cereus* | 7 |
|  |  |  | *S. aureus* | 11 |
|  |  |  | *A. brasiliensis* | 7 |
|  |  |  | *C. albicans* | 7 |
| 50 | Geranylacetone oxime | | *E. coli* | 8 |
|  |  |  | *E. hirae* | 11 |
|  |  |  | *P. aeruginosa* | 7 |
|  |  |  | *B. cereus* | 7 |
|  |  |  | *S. aureus* | 12 |
|  |  |  | *A. brasiliensis* | 7 |
|  |  |  | *C. albicans* | 11 |
| 51 | α-Hexylcinnamaldehyde | | *E. coli* | 7 |
|  |  |  | *E. hirae* | 10 |
|  |  |  | *P. aeruginosa* | 7 |
|  |  |  | *B. cereus* | 9 |
|  |  |  | *S. aureus* | 12 |
|  |  |  | *A. brasiliensis* | 7 |
|  |  |  | *C. albicans* | 7 |
| 52 | α-Hexylcinnamaldehyde oxime | | *E. coli* | 12 |
|  |  |  | *E. hirae* | 14 |
|  |  |  | *P. aeruginosa* | 8 |
|  |  |  | *B. cereus* | 9 |
|  |  |  | *S. aureus* | 11 |
|  |  |  | *A. brasiliensis* | 7 |
|  |  |  | *C. albicans* | 14 |
| 53 | α-Ionone | | *E. coli* | 7 |
|  |  |  | *E. hirae* | 8 |
|  |  |  | *P. aeruginosa* | 7 |
|  |  |  | *B. cereus* | 9 |
|  |  |  | *S. aureus* | 8 |
|  |  |  | *A. brasiliensis* | 7 |
|  |  |  | *C. albicans* | 7 |
| 54 | α-Ionone oxime | | *E. coli* | 7 |
|  |  |  | *E. hirae* | 7 |
|  |  |  | *P. aeruginosa* | 7 |
|  |  |  | *B. cereus* | 9 |
|  |  |  | *S. aureus* | 9 |
|  |  |  | *A. brasiliensis* | 7 |
|  |  |  | *C. albicans* | 7 |
| 54 | β-Ionone | | *E. coli* | 11 |
|  |  |  | *E. hirae* | 10 |
|  |  |  | *P. aeruginosa* | 8 |
|  |  |  | *B. cereus* | 12 |
|  |  |  | *S. aureus* | 13 |
|  |  |  | *A. brasiliensis* | 8 |
|  |  |  | *C. albicans* | 7 |
| 56 | β-Ionone oxime | | *E. coli* | 8 |
|  |  |  | *E. hirae* | 7 |
|  |  |  | *P. aeruginosa* | 8 |
|  |  |  | *B. cereus* | 8 |
|  |  |  | *S. aureus* | 9 |
|  |  |  | *A. brasiliensis* | 7 |
|  |  |  | *C. albicans* | 7 |
| 57 | α -Isomethyl ionone | | *E. coli* | 7 |
|  |  |  | *E. hirae* | 7 |
|  |  |  | *P. aeruginosa* | 7 |
|  |  |  | *B. cereus* | 9 |
|  |  |  | *S. aureus* | 8 |
|  |  |  | *A. brasiliensis* | 7 |
|  |  |  | *C. albicans* | 7 |
| 58 | α -Isomethyl ionone oxime | | *E. coli* | 10 |
|  |  |  | *E. hirae* | 15 |
|  |  |  | *P. aeruginosa* | 7 |
|  |  |  | *B. cereus* | 9 |
|  |  |  | *S. aureus* | 8 |
|  |  |  | *A. brasiliensis* | 7 |
|  |  |  | *C. albicans* | 15 |
| 59 | Isophorone | | *E. coli* | 8 |
|  |  |  | *E. hirae* | 8 |
|  |  |  | *P. aeruginosa* | 7 |
|  |  |  | *B. cereus* | 9 |
|  |  |  | *S. aureus* | 10 |
|  |  |  | *A. brasiliensis* | 8 |
|  |  |  | *C. albicans* | 7 |
| 60 | Isophorone oxime | | *E. coli* | 9 |
|  |  |  | *E. hirae* | 12 |
|  |  |  | *P. aeruginosa* | 7 |
|  |  |  | *B. cereus* | 7 |
|  |  |  | *S. aureus* | 7 |
|  |  |  | *A. brasiliensis* | 7 |
|  |  |  | *C. albicans* | 12 |
| 61 | *cis*-Jasmone | | *E. coli* | 8 |
|  |  |  | *E. hirae* | 11 |
|  |  |  | *P. aeruginosa* | 7 |
|  |  |  | *B. cereus* | 10 |
|  |  |  | *S. aureus* | 11 |
|  |  |  | *A. brasiliensis* | 13 |
|  |  |  | *C. albicans* | 9 |
| 62 | *cis*-Jasmone oxime | | *E. coli* | 8 |
|  |  |  | *E. hirae* | 13 |
|  |  |  | *P. aeruginosa* | 7 |
|  |  |  | *B. cereus* | 7 |
|  |  |  | *S. aureus* | 9 |
|  |  |  | *A. brasiliensis* | 13 |
|  |  |  | *C. albicans* | 13 |
| 63 | (+)-Menthone oxime | | *E. coli* | 9 |
|  |  |  | *E. hirae* | 7 |
|  |  |  | *P. aeruginosa* | 7 |
|  |  |  | *B. cereus* | 7 |
|  |  |  | *S. aureus* | 7 |
|  |  |  | *A. brasiliensis* | 7 |
|  |  |  | *C. albicans* | 7 |
| 64 | ( ̶ )-Menthone | | *E. coli* | 7 |
|  |  |  | *E. hirae* | 8 |
|  |  |  | *P. aeruginosa* | 7 |
|  |  |  | *B. cereus* | 8 |
|  |  |  | *S. aureus* | 18 |
|  |  |  | *A. brasiliensis* | 7 |
|  |  |  | *C. albicans* | 8 |
| 65 | ( ̶ )-Menthone oxime | | *E. coli* | 10 |
|  |  |  | *E. hirae* | 7 |
|  |  |  | *P. aeruginosa* | 8 |
|  |  |  | *B. cereus* | 8 |
|  |  |  | *S. aureus* | 7 |
|  |  |  | *A. brasiliensis* | 7 |
|  |  |  | *C. albicans* | 7 |
| 66 | Methyl jasmonate | | *E. coli* | 7 |
|  |  |  | *E. hirae* | 10 |
|  |  |  | *P. aeruginosa* | 7 |
|  |  |  | *B. cereus* | 8 |
|  |  |  | *S. aureus* | 10 |
|  |  |  | *A. brasiliensis* | 10 |
|  |  |  | *C. albicans* | 12 |
| 67 | Methyl jasmonate oxime | | *E. coli* | 9 |
|  |  |  | *E. hirae* | 8 |
|  |  |  | *P. aeruginosa* | 7 |
|  |  |  | *B. cereus* | 7 |
|  |  |  | *S. aureus* | 7 |
|  |  |  | *A. brasiliensis* | 7 |
|  |  |  | *C. albicans* | 10 |
| 68 | α-Methyl-*trans*-cinnamaldehyde | | *E. coli* | 7 |
|  |  |  | *E. hirae* | 8 |
|  |  |  | *P. aeruginosa* | 7 |
|  |  |  | *B. cereus* | 9 |
|  |  |  | *S. aureus* | 11 |
|  |  |  | *A. brasiliensis* | 9 |
|  |  |  | *C. albicans* | 7 |
| 69 | α-Methyl-*trans*-cinnamaldehyde oxime | | *E. coli* | 7 |
|  |  |  | *E. hirae* | 10 |
|  |  |  | *P. aeruginosa* | 8 |
|  |  |  | *B. cereus* | 9 |
|  |  |  | *S. aureus* | 8 |
|  |  |  | *A. brasiliensis* | 7 |
|  |  |  | *C. albicans* | 10 |
| 70 | ( ̶ )-Myrtenal | | *E. coli* | 11 |
|  |  |  | *E. hirae* | 12 |
|  |  |  | *P. aeruginosa* | 7 |
|  |  |  | *B. cereus* | 8 |
|  |  |  | *S. aureus* | 8 |
|  |  |  | *A. brasiliensis* | 7 |
|  |  |  | *C. albicans* | 9 |
| 71 | ( ̶ )-Myrtenal oxime | | *E. coli* | 7 |
|  |  |  | *E. hirae* | 7 |
|  |  |  | *P. aeruginosa* | 7 |
|  |  |  | *B. cereus* | 11 |
|  |  |  | *S. aureus* | 11 |
|  |  |  | *A. brasiliensis* | 7 |
|  |  |  | *C. albicans* | 7 |
| 72 | ( ̶ )-Perillaldehyde | | *E. coli* | 7 |
|  |  |  | *E. hirae* | 9 |
|  |  |  | *P. aeruginosa* | 7 |
|  |  |  | *B. cereus* | 7 |
|  |  |  | *S. aureus* | 19 |
|  |  |  | *A. brasiliensis* | 9 |
|  |  |  | *C. albicans* | 7 |
| 73 | ( ̶ )-Perillaldehyde oxime | | *E. coli* | 9 |
|  |  |  | *E. hirae* | 10 |
|  |  |  | *P. aeruginosa* | 7 |
|  |  |  | *B. cereus* | 7 |
|  |  |  | *S. aureus* | 7 |
|  |  |  | *A. brasiliensis* | 8 |
|  |  |  | *C. albicans* | 10 |
| 74 | Phenylacetaldehyde | | *E. coli* | 7 |
|  |  |  | *E. hirae* | 9 |
|  |  |  | *P. aeruginosa* | 10 |
|  |  |  | *B. cereus* | 10 |
|  |  |  | *S. aureus* | 11 |
|  |  |  | *A. brasiliensis* | 8 |
|  |  |  | *C. albicans* | 10 |
| 75 | Phenylacetaldehyde oxime | | *E. coli* | 10 |
|  |  |  | *E. hirae* | 10 |
|  |  |  | *P. aeruginosa* | 7 |
|  |  |  | *B. cereus* | 7 |
|  |  |  | *S. aureus* | 7 |
|  |  |  | *A. brasiliensis* | 7 |
|  |  |  | *C. albicans* | 8 |
| 76 | 1-Phenylpropan-2-on | | *E. coli* | 7 |
|  |  |  | *E. hirae* | 10 |
|  |  |  | *P. aeruginosa* | 10 |
|  |  |  | *B. cereus* | 7 |
|  |  |  | *S. aureus* | 12 |
|  |  |  | *A. brasiliensis* | 9 |
|  |  |  | *C. albicans* | 7 |
| 77 | 1-Phenylpropan-2-on oxime | | *E. coli* | 8 |
|  |  |  | *E. hirae* | 10 |
|  |  |  | *P. aeruginosa* | 7 |
|  |  |  | *B. cereus* | 8 |
|  |  |  | *S. aureus* | 7 |
|  |  |  | *A. brasiliensis* | 7 |
|  |  |  | *C. albicans* | 10 |
| 78 | 2-Phenylpropionaldehyde | | *E. coli* | 17 |
|  |  |  | *E. hirae* | 11 |
|  |  |  | *P. aeruginosa* | 11 |
|  |  |  | *B. cereus* | 12 |
|  |  |  | *S. aureus* | 11 |
|  |  |  | *A. brasiliensis* | 12 |
|  |  |  | *C. albicans* | 9 |
| 79 | 2-Phenylpropionaldehyde oxime | | *E. coli* | 7 |
|  |  |  | *E. hirae* | 8 |
|  |  |  | *P. aeruginosa* | 7 |
|  |  |  | *B. cereus* | 7 |
|  |  |  | *S. aureus* | 7 |
|  |  |  | *A. brasiliensis* | 17 |
|  |  |  | *C. albicans* | 8 |
| 80 | Piperitone | | *E. coli* | 7 |
|  |  |  | *E. hirae* | 7 |
|  |  |  | *P. aeruginosa* | 7 |
|  |  |  | *B. cereus* | 8 |
|  |  |  | *S. aureus* | 10 |
|  |  |  | *A. brasiliensis* | 10 |
|  |  |  | *C. albicans* | 7 |
| 81 | Piperitone oxime | | *E. coli* | 7 |
|  |  |  | *E. hirae* | 13 |
|  |  |  | *P. aeruginosa* | 7 |
|  |  |  | *B. cereus* | 7 |
|  |  |  | *S. aureus* | 14 |
|  |  |  | *A. brasiliensis* | 7 |
|  |  |  | *C. albicans* | 13 |
| 82 | Piperonal | | *E. coli* | 7 |
|  |  |  | *E. hirae* | 7 |
|  |  |  | *P. aeruginosa* | 7 |
|  |  |  | *B. cereus* | 8 |
|  |  |  | *S. aureus* | 30 |
|  |  |  | *A. brasiliensis* | 15 |
|  |  |  | *C. albicans* | 12 |
| 83 | Piperonal oxime | | *E. coli* | 9 |
|  |  |  | *E. hirae* | 11 |
|  |  |  | *P. aeruginosa* | 7 |
|  |  |  | *B. cereus* | 7 |
|  |  |  | *S. aureus* | 7 |
|  |  |  | *A. brasiliensis* | 7 |
|  |  |  | *C. albicans* | 11 |
| 84 | Propiophenone | | *E. coli* | 7 |
|  |  |  | *E. hirae* | 9 |
|  |  |  | *P. aeruginosa* | 8 |
|  |  |  | *B. cereus* | 7 |
|  |  |  | *S. aureus* | 8 |
|  |  |  | *A. brasiliensis* | 7 |
|  |  |  | *C. albicans* | 7 |
| 85 | Propiophenone oxime | | *E. coli* | 8 |
|  |  |  | *E. hirae* | 14 |
|  |  |  | *P. aeruginosa* | 7 |
|  |  |  | *B. cereus* | 8 |
|  |  |  | *S. aureus* | 7 |
|  |  |  | *A. brasiliensis* | 10 |
|  |  |  | *C. albicans* | 14 |
| 86 | Pseudoionone | | *E. coli* | 7 |
|  |  |  | *E. hirae* | 10 |
|  |  |  | *P. aeruginosa* | 13 |
|  |  |  | *B. cereus* | 21 |
|  |  |  | *S. aureus* | 23 |
|  |  |  | *A. brasiliensis* | 10 |
|  |  |  | *C. albicans* | 10 |
| 87 | Pseudoionone oxime | | *E. coli* | 11 |
|  |  |  | *E. hirae* | 15 |
|  |  |  | *P. aeruginosa* | 10 |
|  |  |  | *B. cereus* | 27 |
|  |  |  | *S. aureus* | 18 |
|  |  |  | *A. brasiliensis* | 7 |
|  |  |  | *C. albicans* | 15 |
| 88 | (+)-Pulegone | | *E. coli* | 7 |
|  |  |  | *E. hirae* | 7 |
|  |  |  | *P. aeruginosa* | 7 |
|  |  |  | *B. cereus* | 8 |
|  |  |  | *S. aureus* | 18 |
|  |  |  | *A. brasiliensis* | 7 |
|  |  |  | *C. albicans* | 8 |
| 89 | (+)-Pulegone oxime | | *E. coli* | 8 |
|  |  |  | *E. hirae* | 7 |
|  |  |  | *P. aeruginosa* | 7 |
|  |  |  | *B. cereus* | 8 |
|  |  |  | *S. aureus* | 11 |
|  |  |  | *A. brasiliensis* | 7 |
|  |  |  | *C. albicans* | 7 |
| 90 | Safranal oxime | | *E. coli* | 8 |
|  |  |  | *E. hirae* | 12 |
|  |  |  | *P. aeruginosa* | 7 |
|  |  |  | *B. cereus* | 9 |
|  |  |  | *S. aureus* | 11 |
|  |  |  | *A. brasiliensis* | 7 |
|  |  |  | *C. albicans* | 12 |
| 91 | *m*-Tolualdehyde | | *E. coli* | 7 |
|  |  |  | *E. hirae* | 8 |
|  |  |  | *P. aeruginosa* | 7 |
|  |  |  | *B. cereus* | 9 |
|  |  |  | *S. aureus* | 10 |
|  |  |  | *A. brasiliensis* | 7 |
|  |  |  | *C. albicans* | 7 |
| 92 | *m*-Tolualdehyde oxime | | *E. coli* | 7 |
|  |  |  | *E. hirae* | 8 |
|  |  |  | *P. aeruginosa* | 11 |
|  |  |  | *B. cereus* | 11 |
|  |  |  | *S. aureus* | 7 |
|  |  |  | *A. brasiliensis* | 10 |
|  |  |  | *C. albicans* | 8 |
| 93 | *o*-Tolualdehyde | | *E. coli* | 7 |
|  |  |  | *E. hirae* | 8 |
|  |  |  | *P. aeruginosa* | 8 |
|  |  |  | *B. cereus* | 9 |
|  |  |  | *S. aureus* | 10 |
|  |  |  | *A. brasiliensis* | 7 |
|  |  |  | *C. albicans* | 7 |
| 94 | *o*-Tolualdehyde oxime | | *E. coli* | 11 |
|  |  |  | *E. hirae* | 7 |
|  |  |  | *P. aeruginosa* | 8 |
|  |  |  | *B. cereus* | 23 |
|  |  |  | *S. aureus* | 7 |
|  |  |  | *A. brasiliensis* | 9 |
|  |  |  | *C. albicans* | 7 |
| 95 | *p*-Tolualdehyde | | *E. coli* | 10 |
|  |  |  | *E. hirae* | 8 |
|  |  |  | *P. aeruginosa* | 7 |
|  |  |  | *B. cereus* | 8 |
|  |  |  | *S. aureus* | 7 |
|  |  |  | *A. brasiliensis* | 7 |
|  |  |  | *C. albicans* | 7 |
| 96 | *p*-Tolualdehyde oxime | | *E. coli* | 7 |
|  |  |  | *E. hirae* | 12 |
|  |  |  | *P. aeruginosa* | 8 |
|  |  |  | *B. cereus* | 7 |
|  |  |  | *S. aureus* | 28 |
|  |  |  | *A. brasiliensis* | 7 |
|  |  |  | *C. albicans* | 12 |
| 97 | Vanillin | | *E. coli* | 7 |
|  |  |  | *E. hirae* | 7 |
|  |  |  | *P. aeruginosa* | 7 |
|  |  |  | *B. cereus* | 8 |
|  |  |  | *S. aureus* | 7 |
|  |  |  | *A. brasiliensis* | 11 |
|  |  |  | *C. albicans* | 7 |
| 98 | Vanillin oxime | | *E. coli* | 8 |
|  |  |  | *E. hirae* | 10 |
|  |  |  | *P. aeruginosa* | 7 |
|  |  |  | *B. cereus* | 7 |
|  |  |  | *S. aureus* | 18 |
|  |  |  | *A. brasiliensis* | 7 |
|  |  |  | *C. albicans* | 10 |
| 99 | Veratraldehyde | | *E. coli* | 7 |
|  |  |  | *E. hirae* | 7 |
|  |  |  | *P. aeruginosa* | 7 |
|  |  |  | *B. cereus* | 9 |
|  |  |  | *S. aureus* | 7 |
|  |  |  | *A. brasiliensis* | 12 |
|  |  |  | *C. albicans* | 7 |
| 100 | Veratraldehyde oxime | | *E. coli* | 8 |
|  |  |  | *E. hirae* | 12 |
|  |  |  | *P. aeruginosa* | 7 |
|  |  |  | *B. cereus* | 7 |
|  |  |  | *S. aureus* | 7 |
|  |  |  | *A. brasiliensis* | 7 |
|  |  |  | *C. albicans* | 13 |
| 101 | | ( ̶ )-Verbenone | *E. coli* | 7 |
|  |  |  | *E. hirae* | 10 |
|  |  |  | *P. aeruginosa* | 7 |
|  |  |  | *B. cereus* | 8 |
|  |  |  | *S. aureus* | 9 |
|  |  |  | *A. brasiliensis* | 7 |
|  |  |  | *C. albicans* | 9 |
| 102 | | ( ̶ )-Verbenone oxime | *E. coli* | 7 |
|  |  |  | *E. hirae* | 7 |
|  |  |  | *P. aeruginosa* | 7 |
|  |  |  | *B. cereus* | 7 |
|  |  |  | *S. aureus* | 7 |
|  |  |  | *A. brasiliensis* | 7 |
|  |  |  | *C. albicans* | 7 |

# TabLE S2. Minimal Inhibitory Concentration of low-molecular carbonyl compounds and THEIR oxime derivatives

| **No** | **Compound** | **Microorganism** | **MIC µg/mL (mM)** |
| --- | --- | --- | --- |
| 1 | α-Amylcinnamaldehyde | *E. hirae* | 1200 (5.52) |
| 2 | α-Amylcinnamaldehyde oxime | *E. hirae* | 150 (0.74) |
|  |  | *C. albicans* | 1200 (5.90) |
|  |  | *L. pneumophila* | 1200 (5.90) |
| 3 | *m*-Anisaldehyde | *E. hirae* | 1200 (8.81) |
|  |  | *S. aureus* | 2400 (17.63) |
| 4 | *m*- Anisaldehyde oxime | *L. pneumophila* | 1200 (9.82) |
|  |  | *S. aureus* | 1200 (9.82) |
| 5 | *o*-Anisaldehyde | *L. pneumophila* | 300 (2.20) |
| 6 | Benzaldehyde | *S. aureus* | 2400 (22.40) |
| 7 | Benzaldehyde oxime | *L. pneumophila* | 1200 (9.91) |
|  |  | *S. aureus* | 1200 (9.91) |
| 8 | (+)-Camphor | *S. aureus* | 1200 (7.83) |
|  |  | *L. pneumophila* | 300 (1.96) |
| 9 | (+)-Camphor oxime | *S. aureus* | 1200 (7.17) |
| 10 | ( ̶ )-Camphor | *E. coli* | 75.5 (0.49) |
|  |  | *E. hirae* | 1200 (7.83) |
|  |  | *L. pneumophila* | 300 (1.96) |
| 11 | ( ̶ )-Camphor oxime | *E. hirae* | 600 (3.59) |
|  |  | *E. coli* | 150 (0.90) |
| 12 | (±)-Camphor oxime | *C. albicans* | 600 (3.59) |
|  |  | *L. pneumophila* | 1200 (7.17) |
| 13 | (+)-Carvone oxime | *E. hirae* | 300 (1.82) |
| 14 | ( ̶ )-Carvone | *C. albicans* | 112.5 (0.74) |
| 15 | ( ̶ )-Carvone oxime | *C. albicans* | 225 (1.36) |
| 16 | 1,8-Cineole | *L. pneumophila* | 600 (4.54) |
|  |  | *E. hirae* | 600 (4.54) |
| 17 | Oxo-1,8-cineole oxime | *L. pneumophila* | 1200 (9.08) |
| 18 | *trans*-Cinnamaldehyde | *E. coli* | 300 (2.27) |
|  |  | *B. cereus* | 600 (4.54) |
|  |  | *C. albicans* | 37.5 (0.28) |
|  |  | *A. brasiliensis* | 112.4 (0.85) |
| 19 | *trans*-Cinnamaldehyde oxime | *E. coli* | 600 (4.08) |
|  |  | *E. hirae* | 2400 (16.31) |
|  |  | *A. brasiliensis* | 37.5 (0.25) |
|  |  | *C. albicans* | 75 (0.51) |
|  |  | *L. pneumophila* | 600 (4.08) |
| 20 | Citral | *P. aeruginosa* | 600 (3.92) |
|  |  | *B. cereus* | 600 (3.92) |
|  |  | *S. aureus* | 1200 (7.83) |
| 21 | Citral oxime | *S. aureus* | 1200 (7.17) |
| 22 | (±)-Citronellal | *E. hirae* | 600 (3.86) |
|  |  | *S. aureus* | 1200 (7.73) |
|  |  | *A. brasiliensis* | 600 (3.86) |
| 23 | (±)-Citronellal oxime | *E. coli* | 300 (1.77) |
|  |  | *A. brasiliensis* | 150 (0.89) |
|  |  | *S. aureus* | 300 (1.77) |
| 24 | β-cyclocitral | *E. hirae* | 1200 (7.83) |
|  |  | *P. aeruginosa* | 300 (1.96) |
|  |  | *L. pneumophila* | 150 (0.98) |
| 25 | (+)-Dihydrocarvone oxime | *E. hirae* | 600 (3.54) |
| 26 | Dihydrocinnamaldehyde | *B. cereus* | 300 (2.22) |
|  |  | *L. pneumophila* | 300 (2.22) |
| 27 | Dihydrocinnamaldehyde oxime | *E. hirae* | 300 (2.01) |
|  |  | *C. albicans* | 75 (0.50) |
| 28 | (±)-Dihydrocitronellal | *E. coli* | 600 (3.77) |
| 29 | (±)-Dihydrocitronellal oxime | *E. coli* | 300 (1.73) |
|  |  | *C. albicans* | 300 (1.73) |
| 30 | Dihydrojasmone | *S. aureus* | 1200 (7.17) |
| 31 | Dihydrojasmone oxime | *E. hirae* | 600 (3.31) |
|  |  | *S. aureus* | 300 (1.65) |
| 32 | Dihydro-α-ionone | *E. coli* | 2400 (12.29) |
|  |  | *E. hirae* | 1200 (6.14) |
|  |  | *P. aeruginosa* | 300 (1.54) |
|  |  | *B. cereus* | 1200 (6.14) |
|  |  | *S. aureus* | 2400 (12.29) |
|  |  | *L. pneumophila* | 2400 (12.29) |
|  |  | *A. brasiliensis* | 2400 (12.29) |
|  |  | *C. albicans* | 2400 (12.29) |
| 33 | Dihydro-α-ionone oxime | *E. coli* | 1200 (5.73) |
|  |  | *L. pneumophila* | 150 (0.72) |
| 34 | Dihydro-β-ionone | *L. pneumophila* | 300 (1.54) |
| 35 | Dihydro-β-ionone oxime | *E. hirae* | 600 (2.87) |
|  |  | *L. pneumophila* | 150 (0.72) |
|  |  | *C. albicans* | 2400 (11.47) |
| 36 | Ethylvanillin | *A. brasiliensis* | 300 (1.79) |
|  |  | *C. albicans* | 150 (0.90) |
|  |  | *L. pneumophila* | 300 (1.79) |
| 37 | Ethylvanillin oxime | *E. hirae* | 600 (3.31) |
|  |  | *C. albicans* | 900 (4.97) |
|  |  | *A. brasiliensis* | 300 (1.66) |
| 38 | (+)-Fenchone | *E. hirae* | 2400 (15.66) |
|  |  | *L. pneumophila* | 1200 (7.83) |
| 39 | (+)-Fenchone oxime | *E. hirae* | 300 (1.79) |
| 40 | (-)-Fenchone | *E. hirae* | 600 (3.92) |
|  |  | *L. pneumophila* | 600 (3.92) |
| 41 | (-)-Fenchone oxime | *E. hirae* | 600 (3.59) |
|  |  | *C. albicans* | 1200 (7.17) |
| 42 | Geranylacetone | *S. aureus* | 1200 (6.14) |
|  |  | *L. pneumophila* | 300 (1.54) |
| 43 | Geranylacetone oxime | *E. hirae* | 300 (1.43) |
|  |  | *S. aureus* | 150 (0.72) |
|  |  | *C. albicans* | 2400 (11.47) |
| 44 | α-Hexylcinnamaldehyde | *E. hirae* | 1200 (5.52) |
|  |  | *S. aureus* | 225 (1.04) |
|  |  | *L. pneumophila* | 300 (1.38) |
| 45 | α-Hexylcinnamaldehyde oxime | *E. coli* | 600 (2.59) |
|  |  | *E. hirae* | 42.18 (0.18) |
|  |  | *C. albicans* | 300 (1.30) |
|  |  | *L. pneumophila* | 1200 (5.19) |
|  |  | *S. aureus* | 37.5 (0.16) |
| 46 | α-Ionone | *L. pneumophila* | 600 (3.10) |
| 47 | α-Ionone oxime | *L. pneumophila* | 112.5 (0.54) |
| 48 | β-Ionone | *E. coli* | 1200 (6.21) |
|  |  | *B. cereus* | 600 (3.10) |
|  |  | *S. aureus* | 1200 (6.21) |
|  |  | *L. pneumophila* | 150 (0.78) |
| 49 | β-Ionone oxime | *L. pneumophila* | 37.5 (0.18) |
|  |  | *S. aureus* | 37.5 (0.18) |
| 50 | α-Isomethyl ionone | *E. hirae* | 1200 (5.79) |
|  |  | *L. pneumophila* | 1200 (5.79) |
| 51 | α-Isomethyl ionone oxime | *E. coli* | 1200 (5.42) |
|  |  | *E. hirae* | 18.8 (0.08) |
|  |  | *C. albicans* | 600 (2.71) |
| 52 | Isophorone oxime | *E. hirae* | 1200 (7.83) |
|  |  | *C. albicans* | 450 (2.94) |
| 53 | *cis*-Jasmone | *E. hirae* | 600 (3.63) |
|  |  | *A. brasiliensis* | 75 (0.45) |
|  |  | *S. aureus* | 1200 (7.26) |
|  |  | *L. pneumophila* | 75 (0.45) |
| 54 | *cis*-Jasmone oxime | *E. hirae* | 600 (3.35) |
|  |  | *A. brasiliensis* | 37.5 (0.21) |
|  |  | *S. aureus* | 300 (1.67) |
|  |  | *C. albicans* | 300 (1.67) |
| 55 | ( ̶ )-Menthone | *S. aureus* | 1200 (7.73) |
| 56 | ( ̶ )-Menthone oxime | *S. aureus* | 900 (5.32) |
| 57 | Methyl jasmonate | *E. hirae* | 600 (2.66) |
|  |  | *C. Albicans* | 1200 (5.33) |
| 58 | α-Methyl-*trans*-cinnamaldehyde | *S. aureus* | 1200 (8.15) |
|  |  | *L. pneumophila* | 300 (2.04) |
| 59 | ( ̶ )-Myrtenal | *E. hirae* | 600 (3.97) |
|  |  | *B. cereus* | 600 (3.97) |
|  |  | *L. pneumophila* | 300 (1.98) |
| 60 | ( ̶ )-Myrtenal oxime | *L. pneumophila* | 600 (3.63) |
|  |  | *B. cereus* | 300 (1.82) |
| 61 | Norcamphor | *E. coli* | 2400 (21.59) |
|  |  | *E. hirae* | 1200 (10.80) |
|  |  | *P. aeruginosa* | 2400 (21.59) |
|  |  | *S. aureus* | 1200 (10.80) |
|  |  | *L. pneumophila* | 2400 (21.59) |
|  |  | *A. brasiliensis* | 2400 (21.59) |
| 62 | Norcamphor oxime | *E. coli* | 2400 (19.17) |
|  |  | *E. hirae* | 1200 (9.59) |
|  |  | *P. aeruginosa* | 2400 (19.17) |
|  |  | *S. aureus* | 1200 (9.59) |
|  |  | *L. pneumophila* | 1200 (9.59) |
|  |  | *A. brasiliensis* | 2400 (19.17) |
| 63 | ( ̶ )-Perillaldehyde | *S. aureus* | 1200 (7.94) |
| 64 | ( ̶ )-Perillaldehyde oxime | *E. hirae* | 600 (3.63) |
|  |  | *S. aureus* | 1200 (7.26) |
| 65 | Phenylacetaldehyde | *S. aureus* | 42.18 (0.35) |
|  |  | *L. pneumophila* | 600 (4.95) |
| 66 | 1-Phenylpropan-2-on | *E. hirae* | 600 (4.44) |
|  |  | *P. aeruginosa* | 2400 (17.75) |
|  |  | *S. aureus* | 1200 (8.88) |
|  |  | *L. pneumophila* | 300 (2.22) |
| 67 | 1-Phenylpropan-2-on oxime | *E. hirae* | 600 (4.02) |
|  |  | *S. aureus* | 2400 (16.09) |
| 68 | 2-Phenylpropionaldehyde | *E. coli* | 600 (4.44) |
|  |  | *E. hirae* | 300 (2.22) |
|  |  | *B. cereus* | 600 (4.44) |
|  |  | *A. brasiliensis* | 37.5 (0.28) |
|  |  | *S. aureus* | 1200 (8.88) |
| 69 | 2-Phenylpropionaldehyde oxime | *L. pneumophila* | 1200 (8.04) |
|  |  | *A. brasiliensis* | 300 (2.01) |
| 70 | Piperitone | *S. aureus* | 1200 (7.83) |
| 71 | Piperitone oxime | *E. hirae* | 600 (3.59) |
|  |  | *S. aureus* | 600 (3.59) |
|  |  | *C. albicans* | 600 (3.59) |
| 72 | Piperonal | *A. brasiliensis* | 112.5 (0.74) |
|  |  | *C. albicans* | 600 (3.97) |
|  |  | *S. aureus* | 2400 (15.88) |
| 73 | Piperonal oxime | *L. pneumophila* | 1200 (7.27) |
|  |  | *S. aureus* | 1200 (7.27) |
|  |  | *C. albicans* | 300 (1.82) |
|  |  | *A. brasiliensis* | 300 (1.82) |
| 74 | Propiophenone | *E. hirae* | 600 (4.44) |
|  |  | *L. pneumophila* | 600 (4.44) |
| 75 | Propiophenone oxime | *E. hirae* | 600 (4.02) |
|  |  | *C. albicans* | 300 (2.01) |
| 76 | Pseudoionone | *E. coli* | 1200 (6.14) |
|  |  | *P. aeruginosa* | 1200 (6.14) |
|  |  | *B. cereus* | 300 (1.54) |
|  |  | *C. albicans* | 112.5 (0.58) |
|  |  | *S. aureus* | 300 (1.54) |
|  |  | *L. pneumophila* | 300 (1.54) |
| 77 | Pseudoionone oxime | *E. coli* | 450 (2.15) |
|  |  | *E. hirae* | 600 (2.87) |
|  |  | *S. aureus* | 37.5 (0.18) |
|  |  | *B. cereus* | 150 (0.72) |
|  |  | *C. albicans* | 37.5 (0.18) |
|  |  | *L. pneumophila* | 150 (0.72) |
| 78 | (+)-Pulegone | *S. aureus* | 1200 (7.09) |
| 79 | (+)-Pulegone oxime | *S. aureus* | 600 (3.27) |
| 80 | Safranal | *E. coli* | 2400 (15.87) |
|  |  | *E. hirae* | 1200 (7.94) |
|  |  | *P. aeruginosa* | 1200 (7.94) |
|  |  | *B. cereus* | 2400 (15.87) |
|  |  | *S. aureus* | 2400 (15.87) |
|  |  | *L. pneumophila* | 2400 (15.87) |
| 81 | Safranal oxime | *E. hirae* | 600 (3.63) |
|  |  | *C. albicans* | 300 (1.82) |
|  |  | *S. aureus* | 150 (0.91) |
| 82 | *m*-Tolualdehyde | *B. cereus* | 1200 (9.90) |
| 83 | *m*-Tolualdehyde oxime | *P. aeruginosa* | 1200 (8.88) |
|  |  | *B. cereus* | 1200 (8.88) |
|  |  | *L. pneumophila* | 1200 (8.88) |
| 84 | *o*-Tolualdehyde | *B. cereus* | 1200 (9.90) |
|  |  | *L. pneumophila* | 600 (4.95) |
| 85 | *o*-Tolualdehyde oxime | *B. cereus* | 1200 (8.88) |
| 86 | *p*-Tolualdehyde | *E. hirae* | 1200 (9.90) |
|  |  | *C. albicans* | 600 (4.95) |
|  |  | *S. aureus* | 2400 (19.81) |
| 87 | *p*-Tolualdehyde oxime | *E. hirae* | 150 (1.11) |
|  |  | *S. aureus* | 1200 (8.88) |
|  |  | *C. albicans* | 300 (2.22) |
| 88 | Vanillin | *A. brasiliensis* | 450 (2.94) |
|  |  | *S. aureus* | 1200 (7.84) |
|  |  | *L. pneumophila* | 300 (1.96) |
| 89 | Vanillin oxime | *S. aureus* | 1200 (7.18) |
|  |  | *A. brasiliensis* | 600 (3.59) |
| 90 | Veratraldehyde | *C. albicans* | 150 (0.90) |
| 91 | Veratraldehyde oxime | *E. hirae* | 600 (3.31) |
|  |  | *C. albicans* | 450 (2.48) |
| 92 | ( ̶ )-Verbenone | *E. coli* | 1200 (7.94) |
|  |  | *L. pneumophila* | 150 (0.99) |
| 93 | ( ̶ )-Verbenone oxime | *L. pneumophila* | 1650 (9.99) |

# TabLE S3. Results OF MTS Assay For all tested low-molecular carbonyl compounds and THEIR oxime derivatives

| **No** | **Compound** | **Viability [%]** | **SD** | **Error [%]** |
| --- | --- | --- | --- | --- |
| 1 | α-Amylcinnamaldehyde | 86.67 | 3.09 | 3.57 |
| 2 | α-Amylcinnamaldehyde oxime | 83.74 | 0.96 | 1.14 |
| 3 | *m*-Anisaldehyde | 100 | 0.70 | 0.69 |
| 4 | *m*-Anisaldehyde oxime | 97.04 | 0.49 | 0.50 |
| 5 | *o-*Anisaldehyde | 100 | 1.37 | 1.29 |
| 6 | *o-*Anisaldehyde oxime | 98.96 | 1.51 | 1.53 |
| 7 | *p*-Anisaldehyde | 100 | 0.67 | 0.66 |
| 8 | *p*-Anisaldehyde oxime | 100 | 0.81 | 0.81 |
| 9 | Benzaldehyde | 100 | 0.67 | 0.62 |
| 10 | Benzaldehyde oxime | 85.64 | 4.01 | 4.68 |
| 11 | (+)-Camphor | 100 | 1.16 | 1.16 |
| 12 | (+)-Camphor oxime | 100 | 7.45 | 7.45 |
| 13 | ( ̶ )-Camphor | 100 | 4.56 | 4.41 |
| 14 | ( ̶ )-Camphor oxime | 92.25 | 1.57 | 1.70 |
| 15 | (±)-Camphor | 100 | 2.78 | 2.64 |
| 16 | (±)-Camphor oxime | 96.32 | 8.98 | 9.32 |
| 17 | (+)-Carvone | 100 | 0.66 | 0.60 |
| 18 | ( ̶ )-Carvone | 100 | 4.51 | 4.31 |
| 19 | ( ̶ )-Carvone oxime | 99.84 | 0.84 | 0.84 |
| 20 | 1.8-Cineole | 98.68 | 2.51 | 2.54 |
| 21 | Oxo-1.8-cineole oxime | 99.61 | 9.65 | 9.69 |
| 22 | *trans-*Cinnamaldehyde | 100 | 0.53 | 0.49 |
| 23 | *trans-*Cinnamaldehyde oxime | 98.69 | 0.06 | 0.06 |
| 24 | Citral | 88.66 | 0.50 | 0.56 |
| 25 | Citral oxime | 100 | 1.36 | 1.36 |
| 26 | β-Cyclocitral | 100 | 0.01 | 0.01 |
| 27 | β-Cyclocitral oxime | 100 | 0.48 | 0.48 |
| 28 | (+)-Dihydrocarvone | 100 | 3.63 | 3.35 |
| 29 | (+)-Dihydrocarvone oxime | 90.71 | 2.71 | 2.99 |
| 30 | Dihydrocinnamaldehyde | 87.57 | 1.55 | 1.77 |
| 31 | Dihydrocinnamaldehyde oxime | 98.63 | 0.24 | 0.24 |
| 32 | Dihydrojasmone | 100 | 6.33 | 5.72 |
| 33 | Dihydrojasmone oxime | 93.58 | 1.86 | 1.99 |
| 34 | Dihydro-β-ionone | 100 | 3.75 | 3.38 |
| 35 | Dihydro-β-ionone oxime | 94.34 | 4.63 | 4.91 |
| 36 | Ethylvanillin | 100 | 1.60 | 1.50 |
| 37 | Ethylvanillin oxime | 100 | 5.95 | 5.95 |
| 38 | (+)-Fenchone | 100 | 8.36 | 8.36 |
| 39 | (+)-Fenchone oxime | 100 | 4.49 | 4.49 |
| 40 | ( ̶ )-Fenchone | 100 | 0.06 | 0.06 |
| 41 | ( ̶ )-Fenchone oxime | 92.99 | 0.46 | 0.49 |
| 42 | Geranylacetone | 100 | 4.71 | 4.15 |
| 43 | Geranylacetone oxime | 92.70 | 0.99 | 1.06 |
| 44 | α-Hexylcinnamaldehyde | 86.64 | 0.37 | 0.43 |
| 45 | α-Hexylcinnamaldehyde oxime | 87.40 | 3.43 | 3.93 |
| 46 | α-Ionone | 100 | 1.85 | 1.69 |
| 47 | α-Ionone oxime | 100 | 0.94 | 0.94 |
| 48 | β-Ionone | 100 | 2.23 | 2.06 |
| 49 | α -Isomethyl ionone | 100 | 2.57 | 2.56 |
| 50 | α -Isomethyl ionone oxime | 87.66 | 1.24 | 1.42 |
| 51 | Isophorone | 100 | 4.76 | 4.41 |
| 52 | Isophorone oxime | 100 | 2.80 | 2.80 |
| 53 | *cis*-Jasmone | 100 | 2.46 | 2.28 |
| 54 | *cis*-Jasmone oxime | 97.42 | 2.76 | 2.83 |
| 55 | (+)-Menthone | 100 | 1.39 | 1.28 |
| 56 | (+)-Menthone oxime | 88.70 | 5.81 | 6.55 |
| 57 | ( ̶ )-Menthone | 100 | 3.10 | 2.99 |
| 58 | ( ̶ )-Menthone oxime | 87.75 | 1.55 | 1.76 |
| 59 | Methyl jasmonate | 100 | 6.59 | 5.17 |
| 60 | Methyl jasmonate oxime | 93.22 | 2.00 | 2.14 |
| 61 | α-Methyl-*trans*-cinnamaldehyde | 93.97 | 0.86 | 0.92 |
| 62 | α-Methyl-*trans*-cinnamaldehyde oxime | 81.89 | 5.20 | 6.35 |
| 63 | ( ̶ )-Myrtenal | 98.76 | 1.70 | 1.73 |
| 64 | ( ̶ )-Myrtenal oxime | 88.44 | 1.68 | 1.90 |
| 65 | Norcamphor | 93.46 | 1.20 | 1.28 |
| 66 | ( ̶ )-Perillaldehyde | 100 | 7.95 | 7.03 |
| 67 | ( ̶ )-Perillaldehyde oxime | 94.61 | 1.92 | 2.03 |
| 68 | Phenylacetaldehyde | 100 | 1.52 | 1.37 |
| 69 | Phenylacetaldehyde oxime | 98.22 | 6.53 | 6.65 |
| 70 | Phenylpropan-2-on | 100 | 3.04 | 3.02 |
| 71 | 1-2-Phenylpropionaldehyde | 98.40 | 2.74 | 2.78 |
| 72 | 1-2-Phenylpropionaldehyde oxime | 95.88 | 1.24 | 1.29 |
| 73 | Piperitone | 100 | 2.53 | 2.35 |
| 74 | Piperitone oxime | 86.93 | 1.72 | 1.98 |
| 75 | Piperonal | 100 | 11.80 | 10.75 |
| 76 | Piperonal oxime | 100 | 1.76 | 1.76 |
| 77 | Propiophenone | 96.12 | 1.99 | 2.07 |
| 78 | Propiophenone oxime | 91.95 | 1.41 | 1.53 |
| 79 | Pseudoionone | 100 | 4.51 | 4.13 |
| 80 | Pseudoionone oxime | 100 | 6.29 | 6.29 |
| 81 | (+)-Pulegone | 100 | 4.78 | 4.30 |
| 82 | (+)-Pulegone oxime | 86.24 | 3.90 | 4.52 |
| 83 | *m*-Tolualdehyde | 100 | 0.61 | 0.55 |
| 84 | *m*-Tolualdehyde oxime | 100 | 1.82 | 1.82 |
| 85 | *o*-Tolualdehyde | 100 | 1.51 | 1.45 |
| 86 | *o*-Tolualdehyde oxime | 100 | 3.26 | 3.26 |
| 87 | *p*-Tolualdehyde | 100 | 0.90 | 0.88 |
| 88 | *p*-Tolualdehyde oxime | 100 | 6.40 | 6.40 |
| 89 | Vanillin | 100 | 0.46 | 0.45 |
| 90 | Vanillin oxime | 100 | 7.27 | 7.27 |
| 91 | Veratraldehyde | 99.87 | 1.79 | 1.79 |
| 92 | Veratraldehyde oxime | 96.16 | 6.89 | 7.17 |
| 93 | ( ̶ )-Verbenone | 95.16 | 4.42 | 4.65 |
| 94 | ( ̶ )-Verbenone oxime | 95.07 | 0.94 | 0.99 |

# TabLE S4. SELECTED ADME-Tox properties for selected COMPOUNDS – PART I.

| No | Compound | MW | SASA | donorHB | accptHB | QPlogPo/w | QPlogHERG | QPPCaco | QPlogBB | QPPMDCK |
| --- | --- | --- | --- | --- | --- | --- | --- | --- | --- | --- |
| 1 | α-Amylcinnamaldehyde | 202.296 | 494.509 | 0.000 | 2.000 | 3.500 | -4.664 | 2172.744 | -0.480 | 1144.501 |
| 2 | α-Amylcinnamaldehyde oxime | 217.310 | 528.028 | 1.000 | 3.200 | 3.115 | -5.176 | 1834.105 | -0.714 | 952.973 |
| 3 | *m-*Anisaldehyde | 136.150 | 338.192 | 0.000 | 2.750 | 1.154 | -3.398 | 1833.764 | -0.154 | 952.781 |
| 4 | *m-*Anisaldehyde oxime | 151.165 | 362.213 | 1.000 | 3.950 | 0.632 | -3.763 | 1520.696 | -0.367 | 778.245 |
| 5 | *o-*Anisaldehyde | 136.150 | 343.345 | 0.000 | 2.750 | 1.192 | -3.556 | 1987.968 | -0.134 | 1039.669 |
| 6 | *o-*Anisaldehyde oxime | 151.165 | 366.015 | 1.000 | 3.950 | 0.668 | -3.875 | 1533.957 | -0.371 | 785.583 |
| 7 | *p-*Anisaldehyde | 136.150 | 337.137 | 0.000 | 2.750 | 1.151 | -3.373 | 1837.754 | -0.151 | 955.022 |
| 8 | *p-*Anisaldehyde oxime | 151.165 | 361.388 | 1.000 | 3.950 | 0.623 | -3.755 | 1522.952 | -0.366 | 779.493 |
| 9 | Benzaldehyde | 106.124 | 300.584 | 0.000 | 2.000 | 1.477 | -3.471 | 1836.895 | -0.078 | 954.539 |
| 10 | Benzaldehyde oxime | 121.138 | 324.156 | 1.000 | 3.200 | 0.988 | -3.828 | 1521.961 | -0.288 | 778.945 |
| 11 | (+)-Camphor | 152.236 | 361.932 | 0.000 | 2.000 | 1.954 | -2.308 | 3958.705 | 0.255 | 2188.942 |
| 12 | (+)-Camphor oxime | 167.250 | 382.900 | 1.000 | 2.700 | 1.663 | -2.526 | 1962.913 | -0.118 | 1025.513 |
| 13 | ( ̶ )-Camphor | 152.236 | 363.034 | 0.000 | 2.000 | 1.954 | -2.353 | 3957.119 | 0.253 | 2187.994 |
| 14 | ( ̶ )-Camphor oxime | 167.250 | 378.818 | 1.000 | 2.700 | 1.647 | -2.471 | 2089.216 | -0.093 | 1097.019 |
| 15 | (±)-Camphor | 152.236 | 361.932 | 0.000 | 2.000 | 1.954 | -2.308 | 3958.705 | 0.255 | 2188.942 |
| 16 | (±)-Camphor oxime | 167.250 | 382.900 | 1.000 | 2.700 | 1.663 | -2.526 | 1962.914 | -0.118 | 1025.514 |
| 17 | (+)-Carvone | 150.220 | 390.374 | 0.000 | 2.000 | 2.159 | -3.312 | 3564.891 | 0.126 | 1954.550 |
| 18 | (+)-Carvone oxime | 165.235 | 411.330 | 1.000 | 2.700 | 1.907 | -3.519 | 2051.664 | -0.213 | 1075.722 |
| 19 | ( ̶ )-Carvone | 150.220 | 390.265 | 0.000 | 2.000 | 2.159 | -3.310 | 3564.994 | 0.126 | 1954.611 |
| 20 | ( ̶ )-Carvone oxime | 165.235 | 411.330 | 1.000 | 2.700 | 1.907 | -3.519 | 2051.925 | -0.213 | 1075.870 |
| 21 | 1.8-Cineole | 154.252 | 377.826 | 0.000 | 0.750 | 2.466 | -2.604 | 9906.038 | 0.606 | 5899.293 |
| 22 | Oxo-1.8-cineole oxime | 181.277 | 398.241 | 1.000 | 2.700 | 2.021 | -2.520 | 2839.227 | 0.010 | 1528.290 |
| 23 | *trans*-Cinnamaldehyde | 132.162 | 354.526 | 0.000 | 2.000 | 1.883 | -4.044 | 1723.030 | -0.261 | 890.747 |
| 24 | *trans*-Cinnamaldehyde oxime | 147.176 | 379.897 | 1.000 | 3.200 | 1.290 | -4.426 | 1320.312 | -0.509 | 668.016 |
| 25 | (±)-Citronellal | 154.252 | 438.680 | 0.000 | 2.000 | 2.503 | -3.545 | 2170.594 | -0.337 | 1143.277 |
| 26 | (±)-Citronellal oxime | 169.266 | 466.393 | 1.000 | 3.200 | 2.016 | -3.870 | 1228.271 | -0.722 | 617.826 |
| 27 | β-Cyclocitral | 152.236 | 380.811 | 0.000 | 2.000 | 2.052 | -2.662 | 3149.900 | 0.100 | 1709.818 |
| 28 | β-Cyclocitral oxime | 167.250 | 401.891 | 1.000 | 3.200 | 1.640 | -2.821 | 1902.865 | -0.210 | 991.647 |
| 29 | (+)-Dihydrocarvone | 154.252 | 385.635 | 0.000 | 2.000 | 2.221 | -2.563 | 3743.465 | 0.164 | 2060.588 |
| 30 | (+)-Dihydrocarvone oxime | 169.266 | 416.200 | 1.000 | 2.700 | 2.092 | -3.006 | 1974.477 | -0.213 | 1032.045 |
| 31 | Dihydrocinnamaldehyde | 134.177 | 362.118 | 0.000 | 2.000 | 2.016 | -3.904 | 1962.838 | -0.213 | 1025.471 |
| 32 | Dihydrocinnamaldehyde oxime | 149.192 | 389.459 | 1.000 | 3.200 | 1.263 | -4.215 | 966.226 | -0.631 | 476.675 |
| 33 | Dihydrojasmone | 166.263 | 446.339 | 0.000 | 2.000 | 2.739 | -3.596 | 3637.526 | -0.078 | 1997.630 |
| 34 | Dihydrojasmone oxime | 181.277 | 458.811 | 1.000 | 2.700 | 2.419 | -3.623 | 2371.765 | -0.370 | 1258.224 |
| 35 | Dihydro-α-ionone | 194.316 | 462.743 | 0.000 | 2.000 | 3.205 | -3.363 | 3550.245 | -0.008 | 1945.871 |
| 36 | Dihydro-α-ionone oxime | 209.331 | 479.467 | 1.000 | 2.700 | 2.811 | -3.471 | 1703.879 | -0.417 | 880.051 |
| 37 | Dihydro-β-ionone | 194.316 | 464.676 | 0.000 | 2.000 | 3.184 | -3.280 | 3602.776 | -0.005 | 1977.010 |
| 38 | Dihydro-β-ionone oxime | 209.331 | 484.001 | 1.000 | 2.700 | 2.801 | -3.436 | 1762.549 | -0.414 | 912.850 |
| 39 | Ethylvanillin | 166.176 | 398.660 | 1.000 | 3.500 | 0.507 | -3.954 | 597.932 | -0.776 | 283.751 |
| 40 | Ethylvanillin oxime | 181.191 | 422.988 | 2.000 | 4.700 | 0.622 | -4.276 | 496.466 | -1.011 | 232.083 |
| 41 | (+)-Fenchone | 152.236 | 396.000 | 0.000 | 2.000 | 2.232 | -3.120 | 4802.891 | 0.292 | 2697.577 |
| 42 | (+)-Fenchone oxime | 167.250 | 380.894 | 1.000 | 2.700 | 1.760 | -2.478 | 2651.445 | -0.011 | 1419.334 |
| 43 | ( ̶ )-Fenchone | 152.236 | 396.000 | 0.000 | 2.000 | 2.232 | -3.120 | 4802.891 | 0.292 | 2697.577 |
| 44 | ( ̶ )-Fenchone oxime | 167.250 | 380.894 | 1.000 | 2.700 | 1.760 | -2.478 | 2651.445 | -0.011 | 1419.334 |
| 45 | Geranylacetone | 194.316 | 527.335 | 0.000 | 2.000 | 3.712 | -4.254 | 3456.831 | -0.256 | 1890.590 |
| 46 | Geranylacetone oxime | 209.331 | 543.484 | 1.000 | 2.700 | 3.326 | -4.315 | 1921.964 | -0.638 | 1002.409 |
| 47 | α-Hexylcinnamaldehyde | 216.322 | 536.088 | 0.000 | 2.000 | 3.901 | -5.122 | 2171.921 | -0.580 | 1144.033 |
| 48 | α-Hexylcinnamaldehyde oxime | 231.337 | 565.148 | 1.000 | 3.200 | 3.524 | -5.440 | 1832.714 | -0.805 | 952.191 |
| 49 | α-Ionone | 192.300 | 465.554 | 0.000 | 2.000 | 3.157 | -3.528 | 3449.171 | 0.032 | 1886.062 |
| 50 | α-Ionone oxime | 207.315 | 484.862 | 1.000 | 2.700 | 2.841 | -3.706 | 2019.025 | -0.310 | 1057.236 |
| 51 | β-Ionone | 192.300 | 482.405 | 0.000 | 2.000 | 3.238 | -3.704 | 3458.932 | -0.045 | 1891.832 |
| 52 | β-Ionone oxime | 207.315 | 501.282 | 1.000 | 2.700 | 2.822 | -3.858 | 1997.224 | -0.400 | 1044.903 |
| 53 | α-Isomethyl ionone | 206.327 | 465.504 | 0.000 | 2.000 | 3.331 | -3.033 | 3614.680 | 0.009 | 1984.072 |
| 54 | α-Isomethyl ionone oxime | 221.342 | 487.723 | 1.000 | 2.700 | 2.984 | -3.336 | 2039.906 | -0.346 | 1069.059 |
| 55 | Isophorone | 138.209 | 363.709 | 0.000 | 2.000 | 1.798 | -2.802 | 3028.600 | 0.145 | 1638.761 |
| 56 | Isophorone oxime | 153.224 | 383.832 | 1.000 | 2.700 | 1.595 | -2.979 | 1651.677 | -0.203 | 850.945 |
| 57 | *cis*-Jasmone | 164.247 | 420.541 | 0.000 | 2.000 | 2.585 | -3.194 | 3692.120 | 0.014 | 2030.056 |
| 58 | *cis*-Jasmone oxime | 179.261 | 446.051 | 1.000 | 2.700 | 2.343 | -3.556 | 2461.563 | -0.282 | 1309.794 |
| 59 | (+)-Menthone | 154.252 | 387.546 | 0.000 | 2.000 | 2.275 | -2.620 | 4325.304 | 0.212 | 2408.844 |
| 60 | (+)-Menthone oxime | 169.266 | 415.338 | 1.000 | 2.700 | 2.111 | -2.972 | 2153.853 | -0.179 | 1133.750 |
| 61 | ( ̶ )-Menthone | 154.252 | 387.873 | 0.000 | 2.000 | 2.276 | -2.630 | 4329.720 | 0.212 | 2411.502 |
| 62 | ( ̶ )-Menthone oxime | 169.266 | 415.328 | 1.000 | 2.700 | 2.111 | -2.972 | 2154.122 | -0.179 | 1133.903 |
| 63 | Methyl jasmonate | 224.299 | 525.072 | 0.000 | 4.000 | 2.412 | -4.096 | 1137.202 | -0.663 | 568.465 |
| 64 | Methyl jasmonate oxime | 239.314 | 533.896 | 1.000 | 4.700 | 2.165 | -4.047 | 659.434 | -1.008 | 315.425 |
| 65 | α-Methyl-*trans*-cinnamaldehyde | 146.188 | 378.476 | 0.000 | 2.000 | 2.115 | -4.025 | 1971.163 | -0.218 | 1030.173 |
| 66 | α-Methyl-*trans*-cinnamaldehyde oxime | 161.203 | 403.474 | 1.000 | 3.200 | 1.605 | -4.385 | 1533.483 | -0.460 | 785.321 |
| 67 | ( ̶ )-Myrtenal | 150.220 | 372.375 | 0.000 | 2.000 | 1.800 | -2.805 | 2036.042 | -0.056 | 1066.871 |
| 68 | ( ̶ )-Myrtenal oxime | 165.235 | 396.966 | 1.000 | 3.200 | 1.479 | -3.186 | 1604.254 | -0.288 | 824.567 |
| 69 | Norcamphor | 110.155 | 305.891 | 0.000 | 2.000 | 0.961 | -2.411 | 3002.670 | 0.156 | 1623.601 |
| 70 | ( ̶ )-Perillaladehyde | 164.247 | 415.021 | 0.000 | 2.000 | 2.410 | -3.397 | 3237.452 | 0.024 | 1761.243 |
| 71 | ( ̶ )-Perillaladehyde oxime | 179.261 | 432.032 | 1.000 | 2.700 | 2.202 | -3.457 | 2012.126 | -0.282 | 1053.332 |
| 72 | Phenylacetaldehyde | 120.151 | 332.112 | 0.000 | 2.000 | 1.667 | -3.663 | 2183.725 | -0.095 | 1150.755 |
| 73 | Phenylacetaldehyde oxime | 135.165 | 358.503 | 1.000 | 3.200 | 1.037 | -3.970 | 1044.547 | -0.512 | 518.572 |
| 74 | 1-Phenylpropan-2-on | 134.177 | 363.622 | 0.000 | 2.000 | 2.032 | -3.941 | 3819.450 | 0.091 | 2105.833 |
| 75 | 1-Phenylpropan-2-on oxime | 149.192 | 383.359 | 1.000 | 2.700 | 2.133 | -4.073 | 1822.882 | -0.315 | 946.671 |
| 76 | 2-Phenylpropionaldehyde | 134.177 | 355.767 | 0.000 | 2.000 | 1.868 | -3.682 | 2570.951 | -0.042 | 1372.818 |
| 77 | 2-Phenylpropionaldehyde oxime | 149.192 | 380.937 | 1.000 | 3.200 | 1.086 | -3.971 | 1327.603 | -0.429 | 672.004 |
| 78 | Piperitone | 152.236 | 395.655 | 0.000 | 2.000 | 2.280 | -3.127 | 4179.425 | 0.186 | 2321.151 |
| 79 | Piperitone oxime | 167.250 | 410.808 | 1.000 | 2.700 | 1.931 | -3.155 | 2200.275 | -0.175 | 1160.185 |
| 80 | Piperonal | 150.134 | 305.559 | 0.000 | 3.500 | 0.492 | -2.588 | 1837.432 | -0.037 | 954.841 |
| 81 | Piperonal oxime | 165.148 | 329.258 | 1.000 | 4.700 | 0.342 | -3.010 | 1524.470 | -0.245 | 780.333 |
| 82 | Propiophenone | 134.177 | 361.536 | 0.000 | 2.000 | 1.849 | -3.917 | 3928.838 | 0.102 | 2171.097 |
| 83 | Propiophenone oxime | 149.192 | 374.084 | 1.000 | 2.700 | 1.953 | -3.908 | 2163.427 | -0.239 | 1139.198 |
| 84 | Pseudoionone | 192.300 | 520.967 | 0.000 | 2.000 | 3.600 | -4.410 | 3148.435 | -0.298 | 1708.958 |
| 85 | Pseudoionone oxime | 207.315 | 540.205 | 1.000 | 2.700 | 3.246 | -4.498 | 1843.060 | -0.664 | 958.003 |
| 86 | (+)-Pulegone | 152.236 | 398.532 | 0.000 | 2.000 | 2.225 | -3.061 | 4330.293 | 0.196 | 2411.848 |
| 87 | (+)-Pulegone oxime | 167.250 | 415.927 | 1.000 | 2.700 | 1.942 | -3.158 | 2155.584 | -0.191 | 1134.735 |
| 88 | Safranal | 152.236 | 380.811 | 0.000 | 2.000 | 2.052 | -2.662 | 3149.900 | 0.100 | 1709.818 |
| 89 | Safranal oxime | 167.250 | 400.115 | 1.000 | 3.200 | 1.637 | -2.755 | 1893.236 | -0.207 | 986.224 |
| 90 | *m-*Tolualdehyde | 120.151 | 333.259 | 0.000 | 2.000 | 1.737 | -3.485 | 1833.035 | -0.101 | 952.372 |
| 91 | *m-*Tolualdehyde oxime | 135.165 | 357.011 | 1.000 | 3.200 | 1.145 | -3.835 | 1521.193 | -0.313 | 778.520 |
| 92 | *o-*Tolualdehyde | 120.151 | 324.604 | 0.000 | 2.000 | 1.751 | -3.360 | 2373.072 | 0.005 | 1258.974 |
| 93 | *o-*Tolualdehyde oxime | 135.165 | 349.643 | 1.000 | 3.200 | 1.130 | -3.730 | 1530.858 | -0.296 | 783.868 |
| 94 | *p-*Tolualdehyde | 120.151 | 332.645 | 0.000 | 2.000 | 1.736 | -3.473 | 1836.425 | -0.099 | 954.275 |
| 95 | *p-*Tolualdehyde oxime | 135.165 | 356.495 | 1.000 | 3.200 | 1.143 | -3.822 | 1521.722 | -0.312 | 778.812 |
| 96 | Vanillin | 158.197 | 360.386 | 1.000 | 5.400 | 0.109 | -2.514 | 953.050 | -0.427 | 469.652 |
| 97 | Vanillin oxime | 173.211 | 394.725 | 2.000 | 6.600 | -0.216 | -3.004 | 551.754 | -0.805 | 260.140 |
| 98 | Veratraldehyde | 181.191 | 400.385 | 1.000 | 4.700 | 0.806 | -3.676 | 1521.913 | -0.439 | 778.918 |
| 99 | ( ̶ )-Verbenone | 150.220 | 368.018 | 0.000 | 2.000 | 1.904 | -2.674 | 3166.247 | 0.166 | 1719.411 |

# TabLE S5. SELECTED ADME-Tox properties for selected COMPOUNDS – PART II.

| No | Compound | QPpolrz | QPlogPC16 | QPlogPoct | QPlogPw | QPlogS | CIQPlogS | QPlogKp | QPlogKhsa | % Oral Absorption |
| --- | --- | --- | --- | --- | --- | --- | --- | --- | --- | --- |
| 1 | α-Amylcinnamaldehyde | 24.373 | 7.724 | 8.617 | 3.117 | -3.419 | -2.801 | -1.450 | 0.193 | 100.00 |
| 2 | α-Amylcinnamaldehyde oxime | 25.025 | 8.454 | 10.635 | 5.577 | -3.287 | -2.718 | -1.338 | -0.007 | 100.00 |
| 3 | *m-*Anisaldehyde | 14.948 | 4.829 | 5.877 | 4.347 | -0.910 | -1.048 | -2.223 | -0.683 | 92.11 |
| 4 | *m-*Anisaldehyde oxime | 15.430 | 5.486 | 7.963 | 6.780 | -1.091 | -1.246 | -2.131 | -0.684 | 87.60 |
| 5 | *o-*Anisaldehyde | 15.273 | 4.858 | 6.280 | 4.397 | -0.959 | -1.048 | -2.122 | -0.665 | 92.96 |
| 6 | *o-*Anisaldehyde oxime | 15.678 | 5.537 | 8.211 | 6.837 | -1.138 | -1.246 | -2.097 | -0.676 | 87.88 |
| 7 | *p-*Anisaldehyde | 14.893 | 4.827 | 5.980 | 4.341 | -0.900 | -1.048 | -2.222 | -0.686 | 92.11 |
| 8 | *p-*Anisaldehyde oxime | 15.364 | 5.471 | 8.024 | 6.775 | -1.081 | -1.246 | -2.131 | -0.687 | 87.56 |
| 9 | Benzaldehyde | 13.057 | 4.499 | 5.237 | 4.114 | -1.112 | -0.759 | -2.122 | -0.662 | 94.02 |
| 10 | Benzaldehyde oxime | 13.521 | 5.152 | 7.078 | 6.544 | -0.841 | -0.963 | -2.032 | -0.708 | 89.69 |
| 11 | (+)-Camphor | 17.914 | 4.589 | 6.407 | 2.846 | -1.990 | -1.409 | -2.294 | -0.171 | 100.00 |
| 12 | (+)-Camphor oxime | 18.199 | 5.306 | 8.102 | 4.828 | -1.999 | -1.593 | -2.694 | -0.208 | 95.62 |
| 13 | ( ̶ )-Camphor | 17.914 | 4.589 | 6.407 | 2.846 | -1.990 | -1.409 | -2.294 | -0.171 | 100.00 |
| 14 | ( ̶ )-Camphor oxime | 18.199 | 5.306 | 8.102 | 4.828 | -1.999 | -1.593 | -2.694 | -0.208 | 95.62 |
| 15 | (±)-Camphor | 17.914 | 4.589 | 6.407 | 2.846 | -1.990 | -1.409 | -2.294 | -0.171 | 100.00 |
| 16 | (±)-Camphor oxime | 18.199 | 5.306 | 8.102 | 4.828 | -1.999 | -1.593 | -2.694 | -0.208 | 95.62 |
| 17 | (+)-Carvone | 19.026 | 4.968 | 6.907 | 3.182 | -2.070 | -1.501 | -2.050 | -0.133 | 100.00 |
| 18 | (+)-Carvone oxime | 19.334 | 5.712 | 8.474 | 5.149 | -2.355 | -1.690 | -2.308 | -0.193 | 100.00 |
| 19 | ( ̶ )-Carvone | 19.021 | 4.968 | 6.906 | 3.181 | -2.069 | -1.501 | -2.050 | -0.133 | 100.00 |
| 20 | ( ̶ )-Carvone oxime | 19.334 | 5.712 | 8.474 | 5.149 | -2.355 | -1.690 | -2.308 | -0.193 | 100.00 |
| 21 | 1.8-Cineole | 18.782 | 4.330 | 5.491 | 1.367 | -3.034 | -3.658 | -0.888 | 0.226 | 100.00 |
| 22 | Oxo-1.8-cineole oxime | 19.772 | 5.584 | 8.563 | 4.750 | -2.301 | -1.856 | -2.382 | -0.106 | 100.00 |
| 23 | *trans*-Cinnamaldehyde | 15.920 | 5.439 | 6.098 | 3.958 | -1.593 | -1.311 | -1.914 | -0.460 | 95.89 |
| 24 | *trans*-Cinnamaldehyde oxime | 16.478 | 6.162 | 8.066 | 6.434 | -1.367 | -1.401 | -1.881 | -0.564 | 90.36 |
| 25 | (±)-Citronellal | 19.269 | 5.365 | 6.613 | 2.202 | -2.669 | -1.421 | -2.276 | -0.061 | 100.00 |
| 26 | (±)-Citronellal oxime | 19.663 | 6.076 | 8.654 | 4.651 | -2.524 | -1.441 | -2.565 | -0.232 | 94.04 |
| 27 | β-Cyclocitral | 18.347 | 4.739 | 6.578 | 2.760 | -2.391 | -1.479 | -2.381 | -0.137 | 100.00 |
| 28 | β-Cyclocitral oxime | 18.811 | 5.504 | 8.536 | 5.193 | -2.134 | -1.504 | -2.604 | -0.258 | 95.24 |
| 29 | (+)-Dihydrocarvone | 19.020 | 4.893 | 6.728 | 2.700 | -2.488 | -1.457 | -2.245 | -0.087 | 100.00 |
| 30 | (+)-Dihydrocarvone oxime | 19.606 | 5.554 | 8.496 | 4.685 | -2.426 | -1.636 | -2.593 | -0.127 | 100.00 |
| 31 | Dihydrocinnamaldehyde | 16.334 | 5.376 | 6.035 | 3.733 | -1.580 | -1.354 | -1.909 | -0.409 | 100.00 |
| 32 | Dihydrocinnamaldehyde oxime | 16.861 | 6.141 | 8.146 | 6.225 | -1.528 | -1.437 | -2.315 | -0.512 | 87.77 |
| 33 | Dihydrojasmone | 20.341 | 5.242 | 7.044 | 2.234 | -2.977 | -1.846 | -1.960 | 0.017 | 100.00 |
| 34 | Dihydrojasmone oxime | 20.261 | 5.926 | 8.548 | 4.129 | -2.708 | -2.000 | -2.119 | -0.099 | 100.00 |
| 35 | Dihydro-α-ionone | 23.944 | 6.258 | 8.247 | 2.660 | -3.451 | -2.372 | -1.994 | 0.258 | 100.00 |
| 36 | Dihydro-α-ionone oxime | 24.075 | 7.040 | 10.038 | 4.657 | -3.237 | -2.477 | -2.414 | 0.147 | 100.00 |
| 37 | Dihydro-β-ionone | 23.719 | 6.048 | 8.151 | 2.473 | -3.487 | -2.372 | -2.079 | 0.261 | 100.00 |
| 38 | Dihydro-β-ionone oxime | 23.888 | 6.801 | 9.910 | 4.454 | -3.317 | -2.477 | -2.491 | 0.152 | 100.00 |
| 39 | Ethylvanillin | 17.207 | 5.766 | 8.670 | 6.342 | -1.548 | -1.649 | -3.112 | -0.489 | 79.61 |
| 40 | Ethylvanillin oxime | 17.702 | 6.487 | 10.651 | 8.777 | -1.563 | -1.589 | -3.018 | -0.661 | 78.84 |
| 41 | (+)-Fenchone | 19.242 | 4.385 | 6.671 | 2.868 | -2.192 | -1.409 | -2.131 | -0.075 | 100.00 |
| 42 | (+)-Fenchone oxime | 18.117 | 5.217 | 7.992 | 4.729 | -2.007 | -1.593 | -2.440 | -0.213 | 100.00 |
| 43 | ( ̶ )-Fenchone | 19.242 | 4.385 | 6.671 | 2.868 | -2.192 | -1.409 | -2.131 | -0.075 | 100.00 |
| 44 | ( ̶ )-Fenchone oxime | 18.117 | 5.217 | 7.992 | 4.729 | -2.007 | -1.593 | -2.440 | -0.213 | 100.00 |
| 45 | Geranylacetone | 25.162 | 6.700 | 8.456 | 2.109 | -4.186 | -2.272 | -1.756 | 0.364 | 100.00 |
| 46 | Geranylacetone oxime | 25.225 | 7.440 | 10.172 | 4.038 | -3.895 | -2.376 | -2.063 | 0.221 | 100.00 |
| 47 | α-Hexylcinnamaldehyde | 26.358 | 8.295 | 9.203 | 3.040 | -4.055 | -3.099 | -1.332 | 0.337 | 100.00 |
| 48 | α-Hexylcinnamaldehyde oxime | 27.000 | 9.067 | 11.249 | 5.445 | -3.788 | -2.994 | -1.243 | 0.123 | 100.00 |
| 49 | α-Ionone | 24.351 | 6.145 | 8.443 | 2.902 | -3.667 | -2.329 | -2.112 | 0.283 | 100.00 |
| 50 | α-Ionone oxime | 24.597 | 6.901 | 10.208 | 4.874 | -3.495 | -2.437 | -2.348 | 0.174 | 100.00 |
| 51 | β-Ionone | 24.136 | 6.011 | 8.293 | 2.542 | -3.823 | -2.329 | -2.106 | 0.290 | 100.00 |
| 52 | β-Ionone oxime | 24.357 | 6.780 | 10.015 | 4.511 | -3.529 | -2.437 | -2.357 | 0.172 | 100.00 |
| 53 | α-Isomethyl ionone | 24.613 | 6.369 | 8.551 | 2.471 | -3.503 | -2.627 | -2.077 | 0.326 | 100.00 |
| 54 | α-Isomethyl ionone oxime | 24.754 | 7.036 | 10.233 | 4.451 | -3.384 | -2.715 | -2.350 | 0.209 | 100.00 |
| 55 | Isophorone | 17.544 | 4.491 | 6.613 | 3.134 | -2.062 | -1.246 | -2.416 | -0.218 | 100.00 |
| 56 | Isophorone oxime | 17.830 | 5.233 | 8.074 | 5.099 | -1.947 | -1.466 | -2.728 | -0.258 | 93.88 |
| 57 | *cis*-Jasmone | 20.049 | 5.308 | 7.132 | 2.507 | -2.613 | -1.803 | -1.981 | -0.020 | 100.00 |
| 58 | *cis*-Jasmone oxime | 20.366 | 5.944 | 8.655 | 4.449 | -2.646 | -1.961 | -2.110 | -0.103 | 100.00 |
| 59 | (+)-Menthone | 19.067 | 4.825 | 6.675 | 2.659 | -2.490 | -1.457 | -2.123 | -0.083 | 100.00 |
| 60 | (+)-Menthone oxime | 19.615 | 5.546 | 8.476 | 4.656 | -2.414 | -1.636 | -2.519 | -0.125 | 100.00 |
| 61 | ( ̶ )-Menthone | 19.073 | 4.820 | 6.675 | 2.660 | -2.490 | -1.457 | -2.122 | -0.083 | 100.00 |
| 62 | ( ̶ )-Menthone oxime | 19.615 | 5.546 | 8.476 | 4.656 | -2.414 | -1.636 | -2.519 | -0.125 | 100.00 |
| 63 | Methyl jasmonate | 25.941 | 7.083 | 10.142 | 4.580 | -3.259 | -2.021 | -2.822 | -0.112 | 95.76 |
| 64 | Methyl jasmonate oxime | 25.736 | 7.768 | 11.757 | 6.483 | -3.212 | -2.292 | -3.090 | -0.109 | 90.08 |
| 65 | α-Methyl-*trans*-cinnamaldehyde | 17.742 | 5.737 | 6.695 | 3.822 | -2.002 | -1.609 | -1.879 | -0.312 | 100.00 |
| 66 | α-Methyl-*trans*-cinnamaldehyde oxime | 18.258 | 6.442 | 8.591 | 6.275 | -2.019 | -1.653 | -1.842 | -0.439 | 93.36 |
| 67 | ( ̶ )-Myrtenal | 17.685 | 4.804 | 6.694 | 3.041 | -2.064 | -1.191 | -2.664 | -0.202 | 96.71 |
| 68 | ( ̶ )-Myrtenal oxime | 18.163 | 5.454 | 8.385 | 5.482 | -1.980 | -1.223 | -2.620 | -0.326 | 92.98 |
| 69 | Norcamphor | 12.359 | 3.127 | 4.627 | 2.797 | -0.967 | -0.515 | -2.527 | -0.574 | 94.81 |
| 70 | ( ̶ )-Perylaladehyde | 20.461 | 5.460 | 7.252 | 3.018 | -2.558 | -1.734 | -2.052 | -0.021 | 100.00 |
| 71 | ( ̶ )-Perylaladehyde oxime | 20.548 | 6.118 | 8.852 | 4.879 | -2.562 | -1.891 | -2.283 | -0.103 | 100.00 |
| 72 | Phenylacetaldehyde | 14.728 | 4.883 | 5.588 | 3.851 | -1.029 | -1.056 | -1.914 | -0.530 | 96.47 |
| 73 | Phenylacetaldehyde oxime | 15.241 | 5.655 | 7.640 | 6.358 | -1.119 | -1.194 | -2.339 | -0.604 | 87.05 |
| 74 | 1-Phenylpropan-2-on | 17.037 | 5.157 | 6.253 | 3.752 | -1.797 | -1.354 | -1.442 | -0.362 | 100.00 |
| 75 | 1-Phenylpropan-2-on oxime | 17.273 | 5.950 | 7.948 | 5.737 | -1.959 | -1.587 | -1.877 | -0.403 | 100.00 |
| 76 | 2-Phenylpropionaldehyde | 16.634 | 5.243 | 6.172 | 3.770 | -1.604 | -1.354 | -1.821 | -0.383 | 100.00 |
| 77 | 2-Phenylpropionaldehyde oxime | 17.054 | 5.970 | 8.188 | 6.245 | -1.719 | -1.437 | -2.185 | -0.486 | 89.20 |
| 78 | Piperitone | 19.296 | 4.852 | 6.947 | 2.920 | -2.268 | -1.543 | -2.033 | -0.090 | 100.00 |
| 79 | Piperitone oxime | 19.278 | 5.551 | 8.436 | 4.825 | -2.355 | -1.728 | -2.405 | -0.166 | 100.00 |
| 80 | Piperonal | 13.886 | 4.849 | 6.078 | 5.145 | -0.153 | -1.112 | -2.359 | -0.956 | 88.25 |
| 81 | Piperonal oxime | 14.344 | 5.423 | 8.084 | 7.576 | -0.533 | -1.367 | -2.268 | -0.812 | 85.92 |
| 82 | Propiophenone | 16.845 | 5.091 | 6.185 | 3.720 | -1.644 | -1.354 | -1.428 | -0.374 | 100.00 |
| 83 | Propiophenone oxime | 16.706 | 5.807 | 7.717 | 5.631 | -1.889 | -1.587 | -1.748 | -0.433 | 100.00 |
| 84 | Pseudoionone | 24.723 | 6.743 | 8.390 | 2.316 | -4.066 | -2.229 | -1.735 | 0.313 | 100.00 |
| 85 | Pseudoionone oxime | 24.868 | 7.462 | 10.077 | 4.210 | -3.836 | -2.337 | -2.011 | 0.180 | 100.00 |
| 86 | (+)-Pulegone | 19.127 | 4.631 | 6.671 | 2.724 | -2.242 | -1.479 | -2.102 | -0.083 | 100.00 |
| 87 | (+)-Pulegone oxime | 19.275 | 5.401 | 8.366 | 4.694 | -2.444 | -1.663 | -2.500 | -0.154 | 100.00 |
| 88 | Safranal | 4.491 | 6.613 | 3.134 | -2.062 | -1.246 | -2.416 | -0.218 | 100.00 | 17.544 |
| 89 | Safranal oxime | 4.491 | 6.613 | 3.134 | -2.062 | -1.246 | -2.416 | -0.218 | 100.00 | 17.544 |
| 90 | *m-*Tolualdehyde | 14.949 | 4.592 | 5.710 | 3.815 | -1.292 | -1.056 | -2.320 | -0.486 | 95.52 |
| 91 | *m-*Tolualdehyde oxime | 15.420 | 5.261 | 7.632 | 6.247 | -1.144 | -1.194 | -2.228 | -0.572 | 90.60 |
| 92 | *o-*Tolualdehyde | 14.729 | 4.663 | 5.556 | 3.828 | -1.246 | -1.056 | -2.042 | -0.514 | 100.00 |
| 93 | *o-*Tolualdehyde oxime | 15.204 | 5.352 | 7.568 | 6.306 | -1.083 | -1.194 | -2.182 | -0.587 | 90.57 |
| 94 | *p-*Tolualdehyde | 14.914 | 4.588 | 5.795 | 3.811 | -1.289 | -1.056 | -2.319 | -0.488 | 95.53 |
| 95 | *p-*Tolualdehyde oxime | 15.392 | 5.258 | 7.642 | 6.242 | -1.140 | -1.194 | -2.229 | -0.573 | 90.59 |
| 96 | Vanillin | 15.357 | 4.982 | 8.773 | 7.437 | -0.827 | -0.530 | -3.208 | -0.788 | 80.90 |
| 97 | Vanillin oxime | 16.212 | 5.650 | 11.689 | 9.896 | -0.988 | -0.571 | -3.477 | -0.848 | 74.75 |
| 98 | Veratraldehyde | 17.643 | 5.961 | 9.069 | 7.073 | -1.296 | -1.531 | -2.198 | -0.651 | 88.63 |
| 99 | ( ̶ )-Verbenone | 18.089 | 4.635 | 6.756 | 3.073 | -2.144 | -1.256 | -2.408 | -0.173 | 100.00 |
